# Functional Diversification of Motor Neuron-specific *Isl1* Enhancers during Evolution

**DOI:** 10.1371/journal.pgen.1005560

**Published:** 2015-10-08

**Authors:** Namhee Kim, Chungoo Park, Yongsu Jeong, Mi-Ryoung Song

**Affiliations:** 1 School of Life Sciences, Cell Dynamics Research Center, Gwangju Institute of Science and Technology, Oryong-dong, Buk-gu, Gwangju, Republic of Korea; 2 School of Biological Sciences and Technology, Chonnam National University, Yongbong-ro, Buk-gu, Gwangju, Republic of Korea; 3 Department of Genetic Engineering, College of Life Sciences and Graduate School of Biotechnology, Kyung Hee University, Yongin-si, Republic of Korea; California Institute of Technology, UNITED STATES

## Abstract

Functional diversification of motor neurons has occurred in order to selectively control the movements of different body parts including head, trunk and limbs. Here we report that transcription of *Isl1*, a major gene necessary for motor neuron identity, is controlled by two enhancers, CREST1 (E1) and CREST2 (E2) that allow selective gene expression of *Isl1* in motor neurons. Introduction of GFP reporters into the chick neural tube revealed that E1 is active in hindbrain motor neurons and spinal cord motor neurons, whereas E2 is active in the lateral motor column (LMC) of the spinal cord, which controls the limb muscles. Genome-wide ChIP-Seq analysis combined with reporter assays showed that Phox2 and the Isl1-Lhx3 complex bind to E1 and drive hindbrain and spinal cord-specific expression of *Isl1*, respectively. Interestingly, Lhx3 alone was sufficient to activate E1, and this may contribute to the initiation of *Isl1* expression when progenitors have just developed into motor neurons. E2 was induced by onecut 1 (OC-1) factor that permits *Isl1* expression in LMCm neurons. Interestingly, the core region of E1 has been conserved in evolution, even in the lamprey, a jawless vertebrate with primitive motor neurons. All E1 sequences from lamprey to mouse responded equally well to Phox2a and the Isl1-Lhx3 complex. Conversely, E2, the enhancer for limb-innervating motor neurons, was only found in tetrapod animals. This suggests that evolutionarily-conserved enhancers permit the diversification of motor neurons.

## Author Summary

During evolution, motor neurons became specialized to control movements of different body parts including head, trunk and limbs. Here we report that two enhancers of *Isl1*, E1 and E2, are active together with transcription factors in motor neurons. Surprisingly, E1 and its response to transcription factors has been conserved in evolution from the lamprey to man, whereas E2 is only found in animals with limbs. Our study provides an evolutionary example of how functional diversification of motor neurons is achieved by a dynamic interplay between enhancers and transcription factors.

## Introduction

Motor neurons are composed of multiple units called motor columns and motor pools specialized to innervate particular peripheral muscles [1]. Motor neurons in the hindbrain innervate and control the movement of head and neck, while somatic motor (sm) neurons in the spinal cord control body muscles. Limbs and body walls are innervated by separate motor columns occupying different mediolateral positions in the ventral spinal cord; thus lateral motor column (LMC) neurons control limbs, preganglionic column (PGC) neurons control sympathetic ganglia, medial motor column (MMC) and hypaxial motor column (HMC, formerly known as MMCl) neurons control the body walls. The diversification of motor neurons is achieved by combinations of transcription factors restricted to particular motor columns: Phox2 factors specify cranial motor neurons and the LIM-homeodomain (HD) factors Isl1 and Lxh3 specify sm neurons [2–5]. Naturally, the interplay between cis-regulatory elements (i.e., promoters and enhancers) and trans-regulatory elements (i.e., transcription factors) is critical for selective gene expression in individual motor neuron subsets. Furthermore, although studies in *Hox* clusters revealed that signals of body patterning initiate motor neuron diversification, motor neuron-specific transcription control of downstream players in action is not still fully understood [6–8].

Isl1 is a member of the LIM-homeodomain (HD) transcription factor family present in somatic and visceral motor (vm) neurons once they are postmitotic [9]. Genetic and biochemical studies have demonstrated that Isl1 is critical for assigning sm neuron identity, and forms a hexamer complex with Lhx3 [5,9]. Once their pan-motor neuronal identity is acquired via the Isl1-Lhx3 complex, motor neurons further diverge to create multiple motor columns. Motor neurons that retain the Isl1-Lhx3 complex become MMC neurons, while the expression of Foxp1 defines LMC and PGC neurons [10,11]. Isl1 continues to be expressed in most somatic and vm neurons, raising the possibility that dynamic transcriptional control of Isl1 is achieved by differences in the cellular environment of the individual motor neuron subsets. Searches for *Isl1* enhancers by comparative functional genomics have revealed multiple cis-regulatory elements (CREs) specific for motor neurons, such as CREST1 and CREST2 identified in zebrafish [12,13]. However, the trans-regulating elements (TREs) that interact with them and the strategy used to achieve accurate spatiotemporal control of subtype-specific enhancer complexes remain unclear.

Interestingly, Isl1 is found in the motor neurons of many animal species, including primitive aquatic animals such as lampreys and ascidians [14,15]. This led us to reason that evolutionary diversification of motor neurons may have occurred along with the transcriptional control of Isl1 activity in newly-defined motor neuronal subsets. Indeed, chordate ascidians contain primitive vm neurons that share molecular characteristics of cranial motor neurons in the vertebrate CNS [14]. Aquatic agnatha (jawless fish) vertebrates such as the lamprey only have sm neurons that contact the body wall, and display traits of MMC and HMC neurons [16,17]. The LMC and PGC neurons arose only later when paired appendages such as limbs (or lateral fins) and a sympathetic nervous system evolved in fish and amphibians [16,18,19]. Thus, motor neurons have constantly developed to expand the repertoire of motor neuron subsets and control novel body parts while the transcriptional control of Isl1 diversified in parallel.

In the present study, we asked whether transcription programs that diversify motor neurons are conserved or change during evolution and, if so, whether motor neurons build new programs when new paired appendages appear. We found that *Isl1* expression in motor neurons was mainly controlled by two enhancers, CREST1 and CREST2 (herein called E1 and E2), with the help of the dedicated transcription factors Phox2, Isl1 and Lhx3, and onecut (OC) factor [12]. Chromatin Immunoprecipitation Sequencing (ChIP-Seq) analysis and reporter assays demonstrated that Phox2, Isl1 and Lxh3 induce E1 activity in motor neurons in the hindbrain and the spinal cord, whereas OC-1 selectively induce E2 activity in limb-innervating motor neurons. Comparative genomic approaches showed that the core region of E1 was conserved from jawless fish to humans, whereas E2 was only found in animals with paired appendages. Together our findings demonstrate that motor neuron-specific expression of Isl1 has been conserved in evolution with the help of two major enhancers, and that new strategies were adopted to accommodate newly added paired appendages.

## Results

### Characterization of *Isl1* enhancers that label motor neuronal subsets

**Fig 1:**
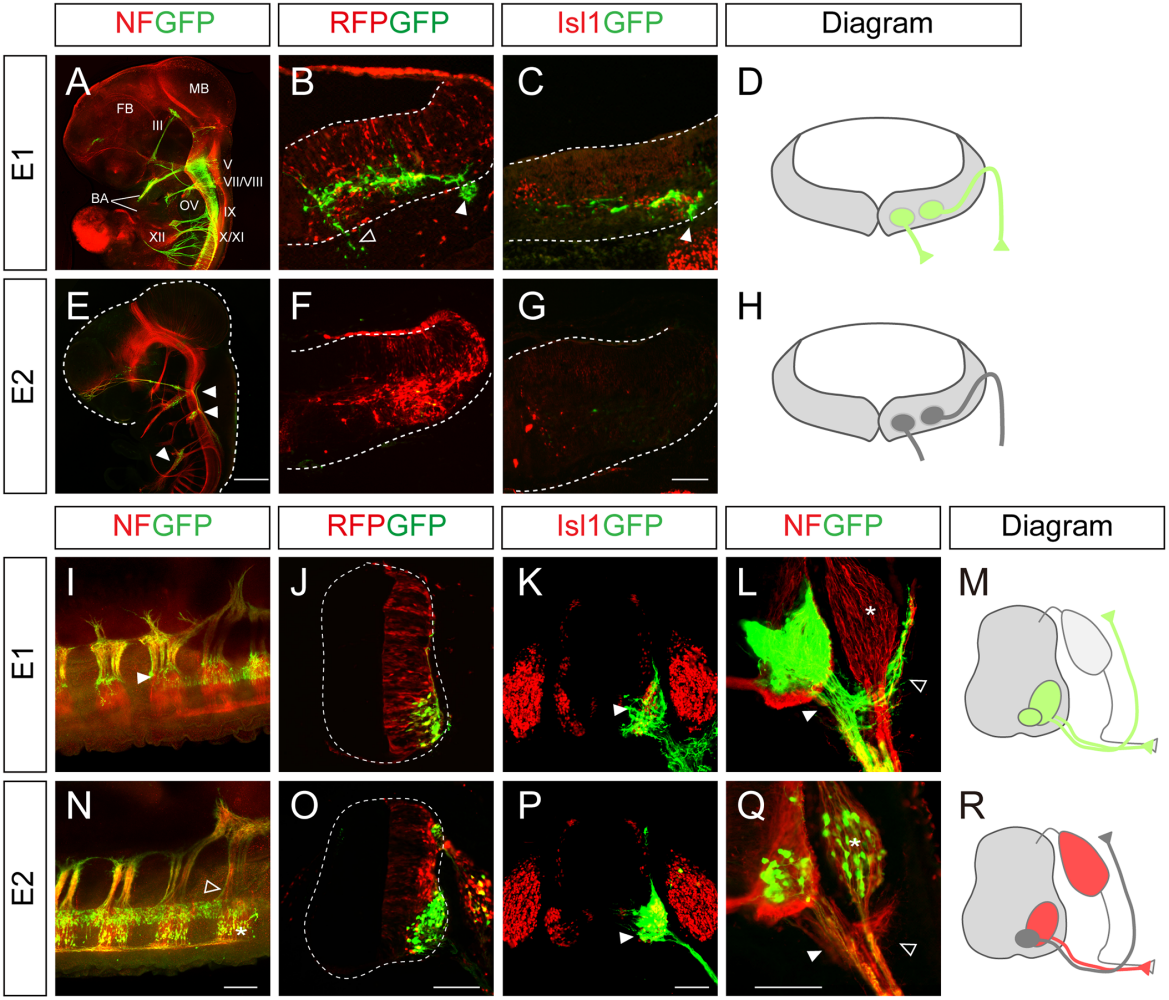
Characterization of the *Isl1* enhancers in the neural tube. (A-C, E-G) GFP expression in wholemount and transverse sections of HH stage 24 chick embryos electroporated with E1::GFP and E2::GFP reporters. The CMV::RFP vector was co-electroporated as an internal control. The E1::GFP reporter labels both branchiomotor (filled arrowheads, B, C) and somatic motor (empty arrowheads, B) axons. E2::GFP weakly labels sensory ganglia (E, arrowheads). FB, forebrain; MB, midbrain; BA, branchial arch; OV, otic vesicle; III, oculomotor N; V, trigeminal N.; VII, facial N.; VIII, abducens N.; IX, glossopharyngeal N.; X, vagus N.; XI, accessory N.; XII, hypoglossal N. (I-L, N-Q) E1::GFP and E2::GFP reporter activity in chick spinal cords after in ovo electroporation. The E1::GFP reporter is active in all somatic motor neurons (arrowheads, I, K, L), including dorsally projecting MMC axons (open arrowhead, L) but not in dorsal root ganglia (DRG, asterisk, L). The E2::GFP reporter is active in ventrally projecting motor neurons (arrowheads, P, Q) and DRG (asterisk, N, Q) but not in MMC neurons (open arrowhead, N, Q) (>20 sections in 8 embryos in each group). (D, H, M, R) Schematic diagrams of the activity of E1 and E2 reporters in the hindbrain and spinal cord. Scale bars: in E, 1 mm for A, E; in G, 100 μm for B, C, F, G; in N, 200 μm for I, N; in O, 100 μm for J, O; in P, 100 μm for K, P; in Q, 100 μm for L, Q.

To understand the mechanism by which Isl1 becomes selectively expressed in certain neuronal subtypes, we chose to characterize two major *Isl1* enhancers originally identified in the zebrafish [12]. To examine their functions in more detail, we generated GFP reporters under the control of the enhancers, and electroporated them into the chick neural tube, aiming at either the hindbrain or spinal cord. There are three different types of motor neurons in the developing hindbrain: branchiomotor (bm), visceral motor (vm) and sm neurons [20]. When the E1::GFP reporter was introduced into the hindbrain, whole mount immunostaining showed that GFP was expressed in the peripheral projections of all types of cranial motor neurons (Fig 1A). Transverse sectioning confirmed that the GFP signal co-distributed with Isl1 immunoreactivity in hindbrain motor neurons (Fig 1B–1D). The mini CMV promoter or minimal *Isl1* promoter produced very low level activity by themselves, confirming the specificity of the E1 enhancer (S1A, S1B, S1D and S1E Fig). An E1 reporter with reverse orientation also showed motor neuron-specific activity, consistent with the orientation-independent character of enhancers (S1C Fig). Conversely, the activity of the E2 GFP reporter was not detectable in motor neurons, but only weakly found in sensory ganglia of the hindbrain (Fig 1E–1H).

In the spinal cord, the distribution of GFP-labeled axons in wholemount immunostained embryos and transverse sections showed that the E1 enhancer was active in all motor columns but not in sensory neurons (Fig 1I–1M). E2 enhancer activity was found in sensory neurons, and LMC and HMC neurons but not in MMC neurons (Fig 1N–1R). 3D reconstruction of z-slice images clearly demonstrated that the E1 but not the E2 reporter was active in MMC neurons (S1 and S2 Movies). Together these results show that E1 is active in motor neurons in both the hindbrain and spinal cord, while E2 activity is restricted to subsets of motor neurons in the spinal cord.

### Spatiotemporal activation of the *Isl1* enhancers occurs in specific motor neuron subtypes

**Fig 2:**
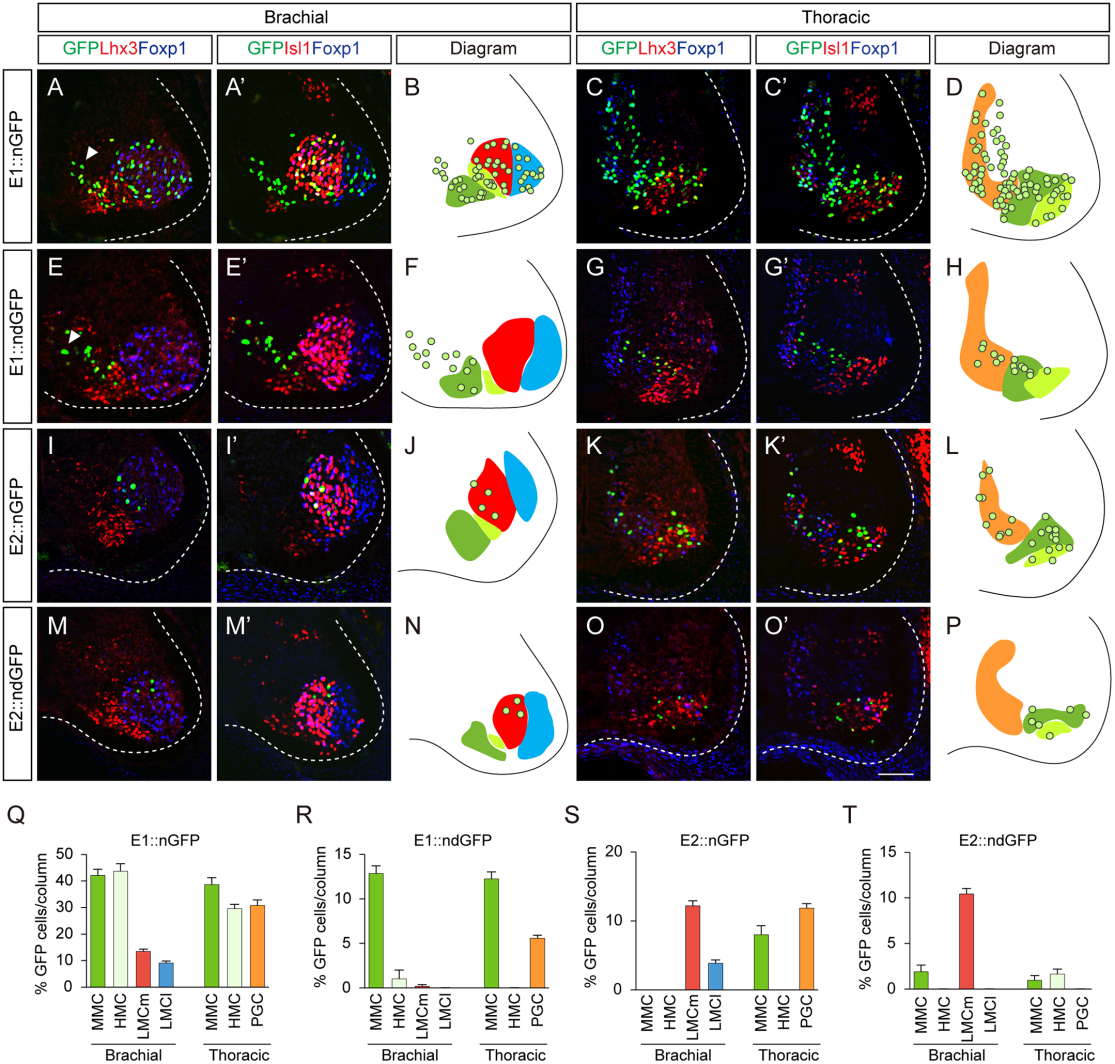
The E1 and E2 enhancers label different subsets of motor neurons. (A-H) Activity of the E1 enhancer fused with the nuclear GFP (nGFP) or destabilized nuclear GFP (ndGFP) reporter electroporated into HH stage 10 to 12 chick neural tubes. The enhancer activity in each motor column was analyzed by quadruple labeling of GFP, Lhx3, Foxp1 and Isl1 by immunohistochemistry. Images of GFP, Lhx3, Foxp1 and GFP, Isl1 and Foxp1 in each condition were obtained from identical sections. Arrowheads are migrating immature motor neurons (A, E). (I-P) Distribution of motor neurons labeled by the E2::nGFP and E2::ndGFP reporters. (B, D, F, H, J, L, N, P) Diagrams summarize the distribution of GFP reporter expression (circles) in MMC (green), LMCm (red), LMCl (blue), HMC (light green) and PGC (orange) at brachial and thoracic levels. (Q-T) The proportion of GFP-expressing cells in each column (>16 sections in 8 embryos in each group) was determined. Error bar represents SEM using three replicates. Scale bar: 50 μm.

To pinpoint the motor somata that were labeled by the E1 and E2 reporters, we constructed reporters for nuclear GFP (nGFP), which becomes localized to cell bodies. Expression of Isl1, Foxp1, Lhx3 and GFP was assessed by quadruple-immunostaining of individual sections to locate individual motor columns. At brachial levels, MMC neurons express Isl1 and Lhx3, while HMC neurons only express Isl1. LMC neurons are divided into medial LMC (LMCm) and lateral LMC (LMCl), which are Foxp1^+^Isl1^+^ and Foxp1^+^Isl1^-^, respectively [10,11]. At these levels, E1 was active in all motor neurons (42.0% in MMC neurons; 43.6% in HMC; 13.4% in LMCm; 9.0% in LMCl) (Fig 2A, 2B and 2Q). This pan-motor neuronal activity of E1, with expression even in the LMCl neurons, which do not express Isl1, led us to reason that stable expression of GFP may persist after the enhancer is no longer active. To monitor enhancer activity *in situ*, we constructed a reporter with destabilized nGFP (ndGFP), whose half-life is less than 4 hours [21]. The majority of cells labeled by destabilized GFP under the control of the E1 enhancer were Lhx3^+^ MMC neurons (12.8%) rather than LMC neurons (0.2%) or HMC neurons (1.0%) (Fig 2E, 2F and 2R). ndGFP expression labeled a streak of cells next to the pMN domain; these were newborn migrating motor neurons (Fig 2E). In contrast, E2::nGFP expression was mostly found in LMC (12.1% in LMCm; 3.9% in LMCl) rather than MMC (0.3%) neurons, and expression of destabilized GFP was mostly found in LMCm neurons (10.4%) and in MMC neurons (1.9%) but not in LMCl neurons, in good agreement with endogenous Isl1 expression in LMCm neurons [22] (Fig 2I, 2J, 2M, 2N, 2S and 2T).

Next we examined the enhancer activities at thoracic levels where Isl1^+^Lhx3^+^ MMC, Isl1^+^Lhx3^-^ HMC and Isl1^+^Foxp1^+^PGC neurons are present [10]. The E1::nGFP reporter was detected in all motor columns (38.6% in MMC; 30.7% in PGC; 29.5% in HMC), whereas the destabilized E1::ndGFP reporter was restricted to MMCs and PGCs and was barely seen in HMCs (Fig 2C, 2D, 2G, 2H, 2Q and 2R). E2::nGFP reporter activity was found in MMCs (8.0%), PGCs (11.9%) and HMCs (1.3%), while the E2::ndGFP reporter was located in PGCs (1.0%) and HMCs (1.6%) (Fig 2K, 2L, 2O, 2P, 2S and 2T). In summary, E1 activity persists in MMC and PGC neurons and E2 is mostly active in LMCm neurons.

### Phox2 homeodomain transcription factors regulate Isl1 expression via the E1 enhancer in bm/vm neurons

We next sought to identify transcription factors in motor neurons that bind to the *Isl1* enhancer. When we looked for potential transcription factor binding sites using rVISTA (http://rvista.dcode.org/), we found multiple transcription factor binding motifs within the E1 enhancer, mostly motifs for homeodomain transcription factors. We then carried out luciferase assays using an E1::luciferase construct cotransduced with diverse homeodomain transcription factors and others that are present in motor neurons as follows: Phox2, Isl1, Lhx3, Barx2, Tbx20, Isl2, Nkx6.1, Hb9, Meis1, Pbx1, Otp, Shox2, Hmx3, Pax6, Sip and OC-1 [23–29]. Of the various homeodomain transcription factors, only Phox2a/b and Lhx3 induced expression of the E1 reporter gene (S2 Fig).

**Fig 3:**
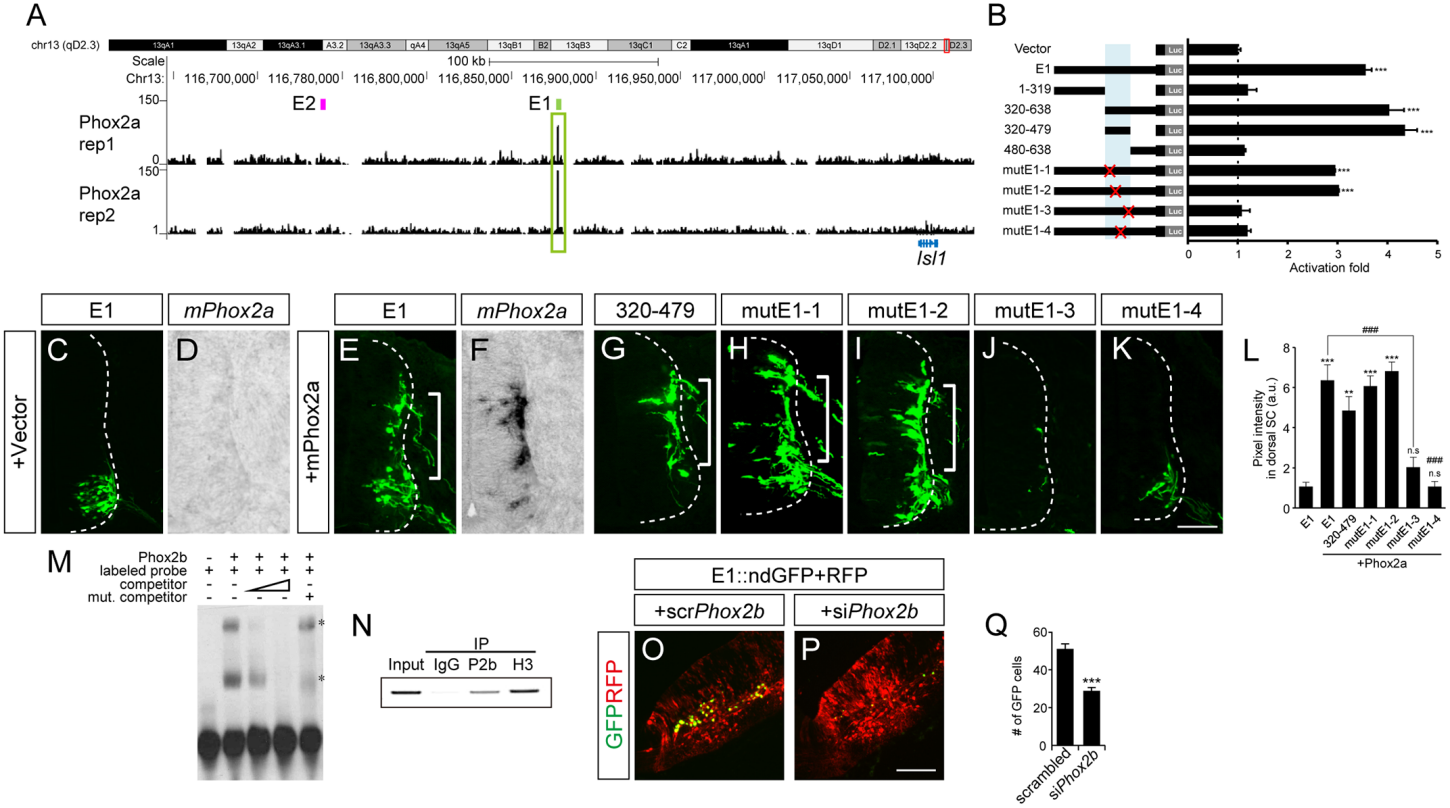
Phox2 regulates Isl1 expression via the E1 enhancer in bm/vm neurons. (A) Phox2a ChIP-Seq peaks around *Isl1* locus in NIP cells from two independent experiments. Note the highest peak in the E1 region. (B) Luciferase reporter activity of the E1 reporter and its derivatives in 293T cells. Error bars represent SEM. ***p < 0.001; unpaired Student’s t-test (n = 3). (C-K) Comparison of E1::GFP reporter derivatives by chick in ovo electroporation. Induction of GFP by Phox2a (brackets, E, G-I) in which *Phox2a* was induced (compare D vs F) was abolished in the case of mutE1-3 and mutE1-4 (J, K). (L) GFP intensity in dorsal spinal cord was measured in each group. Error bars represent SEM. **p < 0.01, ***p < 0.001 vs. E1; ###*p* < 0.001 vs. E1 with Phox2a; n.s., not significant; Kruskal-Wallis test (> 16 sections in 5 embryos in each group). (M, N) Interaction between the Phox2b and E1-4 motifs was demonstrated in gel shift assays and chromatin immunoprecipitation experiments. Asterisks are Phox2-containing protein-DNA complexes. (O, P) E1::ndGFP reporter activity is reduced in si*Phox2b*-electroporated group. CMV::RFP was co-electroporated to confirm electroporation efficiency. (Q) Quantification of E1::ndGFP labeled motor neurons in each group. Error bar represents SEM. ***p < 0.001; unpaired Student’s t-test (n = 34 sections; si*Phox2b*, n = 48 sections). Scale bars: in K, 100 μm for C-K; in P, 100 μm for O, P.

Phox2a and Phox2b are two paralogous homeodomain transcription factors present in branchio-and visceromotor (bm/vm) neurons in the hindbrain [2–4]. Misexpression of Phox2 factors induces ectopic expression of Isl1 when electroporated into the spinal cord, indicating that Phox2 factors may activate *Isl1* enhancers [2,30]. We first explored ChIP-Seq data for Phox2 genomic binding sites in iNIP cells, an inducible embryonic stem cell line with bm/vm neuronal properties [31] (Fig 3A and S3 Fig). Remarkably, the highest Phox2 ChIP-Seq peak between 620 kb downstream and 540 kb upstream of the *Isl1* locus was in E1, around 220 kb downstream of the *Isl1* transcription start site. To further define Phox2 binding sites within E1, a series of deletions and point mutations was introduced into E1 (Fig 3B and S5A Fig). In luciferase assays, Phox2a induced E1 activity by 4.13-fold but did not induce E2, suggesting its specificity to E1 (S2B Fig). A fragment containing nt 320–479 of E1 proved to be sufficient for activation by Phox2a, whereas the mutE1-3 and mutE1-4 sites abolished activation (Fig 3B and S5A Fig).

We and others previously showed that overexpression of Phox2 factors induced ectopic expression of Isl1 within the chick spinal cord, and that the ectopic Isl1^+^ cells had bm/vm characteristics determined by their expression of the bm/vm marker Tbx20 (S4F–S4H and S4K–S4M Fig) [2,30]. The Isl1^+^ cells induced by Phox2a, however, did not express markers for other neuronal classes such as Brn3a (for dI3 neurons) and MNR2 (for sm neurons) (S4I, S4J, S4N and S4O Fig). Thus, we decided to use Isl1^+^ as a phenotypic readout for the ability to induce hindbrain motor neuron identity. When deletions and point mutations were introduced in E1, GFP expression in the spinal cord was not changed except in the cases of nt320-470 E1 and mutE1-3 (S4P–S4V and S4Y Fig). Nt320-470E1 was not active in spinal cord motor neurons because the Isl1-Lhx3 binding motif was partially deleted (S4Q and S5A Figs). Nevertheless it remained active in the hindbrain, implying that the fragment is involved specifically in bm/vm neuron expression (S4X Fig). When Phox2a was electroporated, dorsal expansion of GFP reporter activity was found in nt 320–479 E1, mutE1-1 and mutE1-2, but not in mutE1-3 and mutE1-4 (Fig 3G–3K and S5A Fig). MutE1-3 GFP expression was entirely lost in the neural tube, indicating that the mutE1-3 site is also essential in spinal cord sm neurons (S5A Fig). On the other hand, mutE1-4 only lost its activity in the dorsal spinal cord, in which bm marker Tbx20 but not Brn3a or MNR2 was induced, but its GFP expression was intact in sm neurons (Fig 3K and S4K–S4O Fig). This implies that Phox2 binding to the mutE1-4 motif turns on *Isl1* selectively in hindbrain motor neurons (mostly branchiomotor/visceral) but not in spinal cord motor neurons (mostly somatic). Axonal projection in wholemount and transverse sections of chick embryos also confirmed the restricted activity of mutE1-4 in hindbrain sm neurons (S6A–S6H’ Fig). We also demonstrated specific binding of Phox2 at the E1-4 site by gel shift assays and chromatin IP (Fig 3M and 3N). We next tested whether Phox2 factor is necessary for inducing E1 activity in vivo. When we knocked down chick *Phox2b* by siRNA, E1 activity was diminished in bm neurons (Fig 3O–3Q and S7A Fig). Together, our results suggest that Phox2 factor is necessary and sufficient to induce E1 in hindbrain bm neurons.

**Fig 4:**
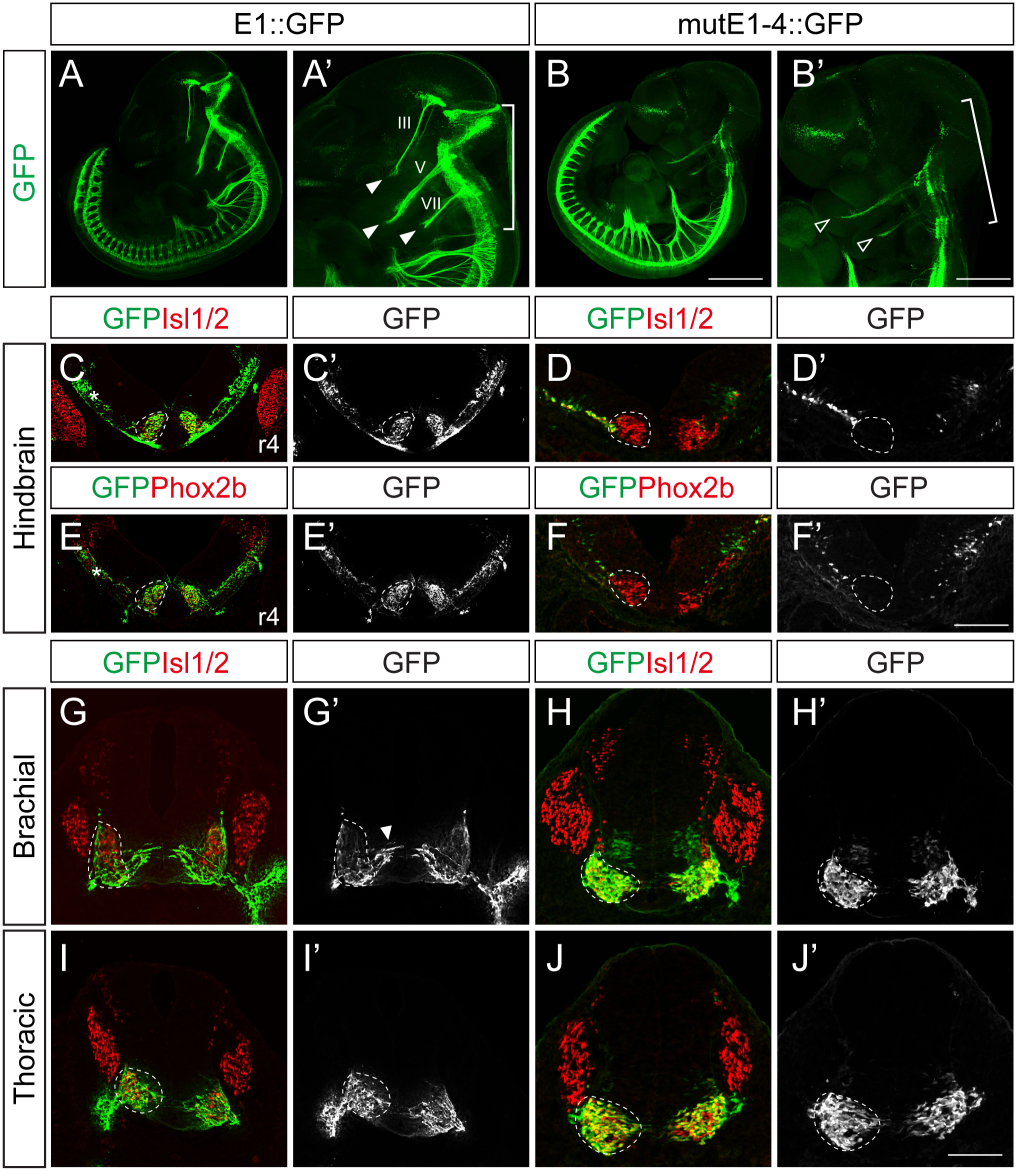
Characterization of E1::GFP and mutE1-4::GFP transgenic mice. (A-B’) Wholemount view of E11.5 E1::GFP and mutE1-4::GFP transgenic mice. In the hindbrain, the branchiomotor (bm) projections were labeled by E1::GFP (bracket, A’) but not by mutE1-4::GFP (bracket, B’). Both the E1 and mutE1-4 reporters are active in the spinal cord. III, V, VII projections are labeled with GFP in E1::GFP mice (filled arrowheads, A’). Only weak expression of V, VII remained in mutE1-4 mice (open arrowheads, B’). (C-F’) Transverse sections of hindbrain r4 facial motor neurons of E11.5 E1::GFP and mutE1-4::GFP transgenic mice. Phox2b^+^ facial motor neurons (dotted circles) express GFP in E1::GFP but not in mutE1-4::GFP mouse embryos. Occasionally, non-specific GFP expression appeared in the dorsal hindbrain area (asterisks, C, E). (G-J’) Transverse sections of the spinal cords of E1::GFP and mutE1-4::GFP transgenic mice. Both mice express GFP in somatic motor (sm) neurons (dotted circles). Arrowhead marks migrating immature motor neurons (G’). Transgenic embryos show more than 80% of consistency in their GFP expression (8/10 in mutE1-4::GFP embryos). Scale bars: in B, 1.2 mm for A, B; in B’, 0.9 mm for A’, B’; in F’, 120 μm for C-F’; in J’, 100 μm for G-J.

Next we generated stable transgenic embryos carrying E1 and mutE1-4 GFP reporters. GFP-labeled peripheral projections were found in the hindbrain and spinal cord of wholemount E1::GFP embryos (Fig 4A–4B’). In transverse sections of these embryos GFP expression was found in Isl1^+^Phox2b^+^ facial motor neurons of rhombomere (r) 4, indicating that E1::GFP is active in bm/vm neurons (Fig 4C, 4C’, 4E and 4E’). Sm neurons in the caudal hindbrain also expressed E1::GFP (S6E, S6G and S6G’ Fig). Wholemount GFP staining of mutE1-4::GFP showed that GFP was expressed in the peripheral projections of the spinal cord but not in the hindbrain (Fig 4B and 4B’). Transverse sections of the hindbrains of mutE1-4::GFP embryos also showed that GFP expression was absent from facial motor neurons and other bm/vm neurons (Fig 4D, 4D’, 4F, 4F’, S6D and S6F Fig). However, the sm neurons in the caudal hindbrain expressed mutE1-4::GFP (S6F, S6H and S6H’ Fig). In the spinal cord, GFP expression by both E1 and mutE1-4 reporters was found in sm neurons at brachial and thoracic levels (Fig 4G–4J’). Thus, Phox2-E1 interaction is required for specific expression of Isl1 in bm/vm neurons.

### Lhx3 and the Isl1-Lhx3 complex activate the E1 enhancer within somatic motor neurons

**Fig 5:**
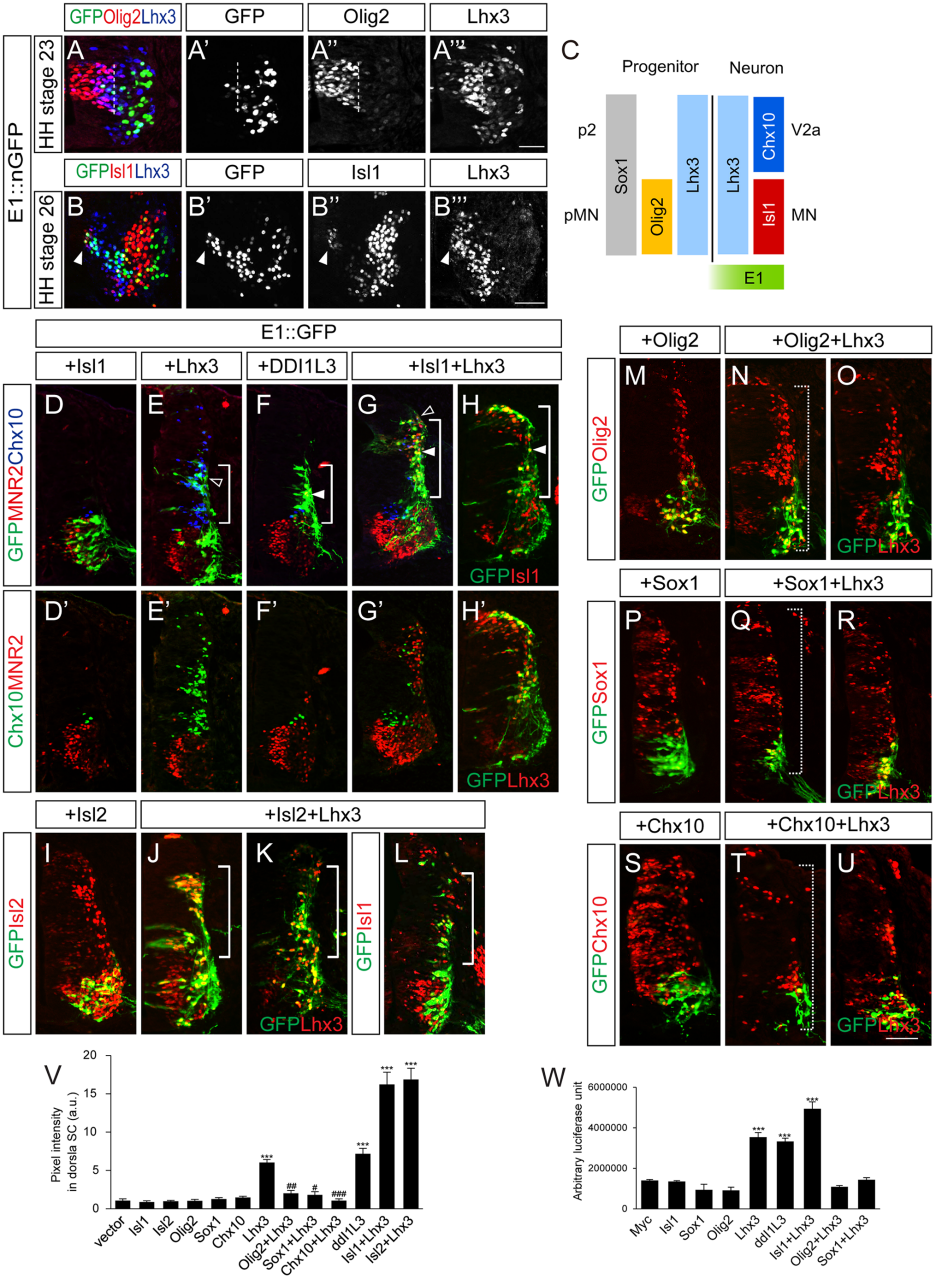
Lhx3 and the Isl1-Lhx3 complex activate the E1 enhancer in somatic motor neurons, and this is repressed by Sox1 and Chx10. (A-B”‘) Activity of the E1::nGFP enhancer and expression of Olig2, Lhx3 or Isl1 in HH stage 23 and 26 chick embryos. Dotted lines mark the border of pMN domain and arrowheads mark migrating motor neurons that weakly express Isl1 (> 16 sections in 6 embryos in each group). (C) Summary diagram of transcription factors present in pMN and p2 progenitors and neurons. (D-H’) Co-electroporation of GFP reporters with Isl1, Lhx3, Isl1 and Lhx3 (Isl1+Lhx3), or ddI1L3 in chick neural tubes. Expression of Isl1 and Lhx3 and their activity to induce ectopic MNR2^+^ motor neurons or Chx10^+^ V2a interneurons were assessed by triple labeling of sections with markers indicated. Lower panels (D’-H’) show identical sections without GFP overlay. The E1 reporter is activated by Lhx3, Isl1+Lhx3 or ddI1L3 (brackets), which overlapped with Chx10 (open arrowheads, E, G) or MNR2 (arrowheads, F-H). (I-L) Ectopic Isl1 expression in the Isl2+Lhx3 group but not in the Lhx3-electroporated group. Overexpression of Isl2 and Lhx3 were confirmed as indicated. (M-U) Co-electroporation of GFP reporters with Lhx3, Olig2, Sox1 or Chx10 as indicated. Overexpression of Olig2, Sox1, Lhx3 and Chx10 were confirmed as indicated. The E1 reporter driven by Lhx3 was repressed in both dorsal and ventral spinal cords in the presence of Olig2, Sox1 and Chx10 (dotted brackets, N, Q, T). (V) GFP intensity in dorsal spinal cord of electroporated groups. Error bars represent SEM. ***p < 0.001 vs. vector; #*p* < 0.05, ##*p* < 0.01, ###*p* < 0.001 vs. Lhx3; Kruskal-Wallis test (> 14 sections in 6 embryos in each group). (W) Induction of the E1 luciferase reporter by various transcription factors. Error bars represent SEM. ***p < 0.001; unpaired Student’s t-test (n = 3). Scale bars: in A””, 20 μm for A-A”‘; in B”‘, 50 μm for B-B”‘; in U, 50 μm for D-U.

Since the E1 enhancer is active in spinal motor neurons and Phox2 is not present in the spinal cord, transcription factors other than Phox2 presumably control its activity in the spinal cord. Our luciferase assays with diverse transcription factors showed that Lhx3 also activated the E1 enhancer (See S2 Fig). Lhx3 is present in p2 and pMN domains and participates in producing V2a interneurons and motor neurons, respectively (Fig 5A–5C) [5]. Isl1 appears when pMN progenitors just become postmitotic, which assigns pan-motor neuron identity by forming a hexamer complex with Lhx3 (Fig 5B and 5C) [5,32]. When motor neurons further diverge into multiple motor columns, only MMC neurons co-express Isl1 and Lhx3 [1,33]. In line with this, we detected E1::ndGFP reporter activity in cells that co-expressed Isl1 and Lhx3 (see Fig 2E, 2F and 2R). To test whether enhancer activity in the spinal cord was altered in the presence of Isl1 and Lhx3, we electroporated cells with the E1::GFP reporter together with Isl1 and Lhx3. When Isl1 alone was electroporated, E1 enhancer activity remained restricted to motor columns, as in the GFP controls (Fig 5D). In contrast, when Lhx3 was electroporated, E1 enhancer activity was expanded into the dorsal column where ectopic Chx10^+^ V2a interneurons arose, although to a lesser degree than the group electroporated with Isl1 and Lhx3 (Fig 5E and 5V). Co-electroporation of Isl1 and Lhx3, or introduction of DDI1L3, which mimics the Isl1-Lhx3 complex, also resulted in expansion of E1 activity in the dorsal spinal cord, in which ectopic MNR2^+^ motor neurons were induced (Fig 5F–5H’ and 5V) [5]. We also tested whether misexpression of LIM factors induced transcription of the endogenous *Isl1* gene in the dorsal spinal cord where the GFP reporter was induced. Since our Isl1 antibody does not distinguish between chick and mouse Isl1 (they are 99% similar), we used Isl2 instead of Isl1, a paralogue of Isl1 with similar biological activity [34,35]. No E1 reporter activity was found in the presence of Isl2 alone (Fig 5I and 5V). In contrast, when Isl2 and Lhx3 were introduced, ectopic Isl1 appeared in the dorsal spinal cord, consistent with the expansion of GFP expression driven by E1 (Fig 5J–5L and 5V). To determine whether the effect of Lhx3 required the LIM domain, we functionally blocked that domain by co-electroporating ΔL-Lhx3, LMO4 or the dimerized domain (DD) [5]. The induction of the E1 enhancer was blocked in all three conditions, indicating that LIM domain-based complex formation is required for Lhx3 to activate the E1 enhancer (S8 Fig). Luciferase reporter assays also showed that E1 was induced by Lhx3, Isl1+Lhx3 and DDI1L3 but not by Isl1 (Fig 5W).

### Repressors prevent abnormal activation of E1 in progenitors and V2 interneurons

Although Lhx3 is present in pMN progenitors and activates E1, we did not observe E1 activity in the pMN region (Fig 5A). Only a streak of migrating motor neuron progenitors that had just left the pMN domain expressed the E1::GFP reporter, and this coincided with the appearance of a low level of Isl1 protein (Fig 5B–5B”‘). Thus, some unknown repressor may suppress the initial expression of Isl1 in pMN progenitors and this repression may be released around the time when the progenitors become postmitotic motor neurons [9]. To test this hypothesis, we tested the effect of Olig2 on Lhx3 expression since Olig2 is present in the pMN domain, is required for motor neuron specification and is extinguished in postmitotic motor neurons [36,37]. In HH stage 23 embryos, differentiating progenitors at lateral region of the pMN domain co-expressed Olig2 and Lhx3 but lacked E1::GFP activity (Fig 5A–5A”‘). As expected, co-electroporation of Olig2 with Lhx3 suppressed the activity of the latter as an inducer of E1 (Fig 5N, 5O and 5V). The induction of E1 luciferase reporter activity by Lhx3 was also inhibited by Olig2 (Fig 5W). Conversely, Olig2 was not effective in reducing E1::GFP expression in endogenous motor neurons or E1 GFP activity driven by Isl1 and Lhx3 (S5C Fig). Thus, Olig2 selectively blocks activity of Lhx3 complex. This was abolished when putative E-box sequence in E1 was mutated, indicating that Olig2 may bind to this site (S5A and S5K–S5M Fig).

Since Lhx3 is also present in the p2 domain where V2 interneurons arise, the E1 enhancer might also be expected to be active in the p2 domain and V2 interneurons. However, this was not observed in our experiments, raising the possibility that an unknown repressor suppresses E1 activity in the p2 domain. Therefore we tested the effect of two known repressors, Sox1 and Chx10, present in the p2 domain and postmitotic V2a interneurons, respectively [38–40]. When electroporated, Sox1 and Chx10 were effective in repressing the ectopic induction of GFP reporter activity driven by Lhx3 (Fig 5Q, 5R, 5T, 5U and 5V). There are two predicted Chx10-binding motifs in E1 but mutating them did not abolish the repressive activity of Chx10 (S5A and S5N–S5S Fig). Since Chx10 binds to AT-rich nucleotides and is known to block hexameric Isl1-Lhx3 at the *Hb9* promoter, we decided to test whether the Isl1-Lhx3 binding motif E1-3 that we had identified was required for repression by Chx10 [32]. Because mutating the E1-3 site abrogated E1 activity, we could not test the repressive activity of Chx10 using mutE1-3. Instead, we used tandem repeats of the E1-3 site (6xE1-3) for GFP reporter and found that its induction by Lhx3 or Isl1-Lhx3 was blocked by Chx10 (S5T–S5V, S5X and S5Y Fig). This appeared to require DNA binding since the Chx10 N51A point mutant defective in DNA binding failed to repress the induction of 6xE1-3 (S5W Fig) [41]. In the case of Sox1, ΔNSox1 was still potent in its repression but C-Sox1 lacking the DNA binding domain was not (S5G–S5J Fig) [40]. Thus, DNA binding is also required for Sox1-mediated repression. The E1 reporter was still active in motor neurons in the presence of Sox1 or Chx10, indicating that the repressors are only effective in non-motor neurons. Nevertheless, Chx10 but not Sox1 was able to inhibit the induction of E1::GFP reporter by exogenous Isl1 and Lhx3 (S5D and S5E Fig). We conclude that E1 activity is repressed by Sox1 in p2 progenitors and by Chx10 in V2a interneurons.

### Isl1 and Lhx3 bind to E1 enhancer in somatic motor neurons

**Fig 6:**
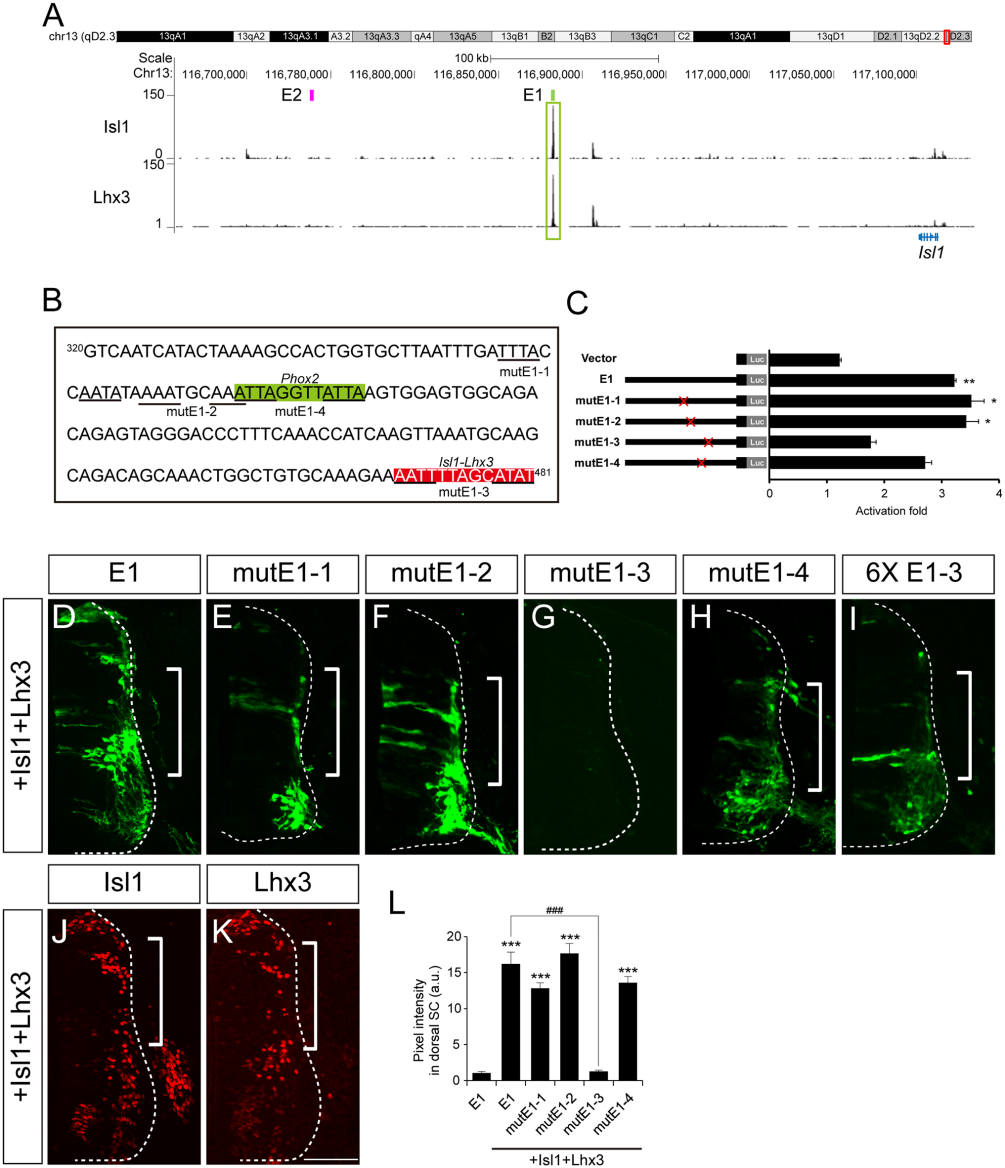
The Isl1-Lhx3 complex activates the E1 enhancer in somatic motor neurons. (A) Isl1 and Lhx3 ChIP-Seq peaks around the *Isl1* locus in NIL cells. The highest peaks of both ChIP-Seq profiles lie in E1. (B) The Phox2 motif (shaded in green) and LIM-HD motif (shaded in red) are shown in E1. The point mutations introduced into the homeodomain recognition motifs are underlined. (C) Luciferase activity of various E1 reporters in the presence of ddI1L3. Error bars represent SEM. *p < 0.05; **p < 0.01; unpaired Student’s t-test (n = 3). (D-K) In ovo electroporation of E1 derivatives with Isl1 and Lhx3 constructs. MutE1-3 is not active in the dorsal spinal cord in the presence of Isl1 and Lhx3, unlike the others (brackets, D-F, H). Six tandem repeats of the E1-3 site (6xE1-3) were sufficient for ectopic reporter activity (bracket, I). Expression of Isl1 and Lhx3 was validated in adjacent sections in which E1 was electroporated (J, K). (L) GFP intensity in dorsal spinal cord was measured in each group. Error bars represent SEM. ***p < 0.001 vs. E1; ###*p* < 0.001 vs. E1 with Isl1+Lhx3; Kruskal-Wallis test (> 14 sections in 5 embryos in each group). Scale bar: 100 μm.

To search for Isl1-Lhx3-binding motifs in the E1 reporter, we examined the results of ChIP-Seq in NIL cells, inducible embryonic stem cells with sm neuronal traits [31]. Strong binding of Isl1 and Lhx3 occurred in E1 (Fig 6A). Of the luciferase reporters with point mutations, all were induced by Isl1 and Lhx3 except mutE1-3, and the activity of all the GFP reporters except mutE1-3 appeared ectopically in the dorsal spinal cord when Isl1 and Lhx3 were co-electroporated (Fig 6B–6H and 6J–6L). E2 was not induced by Isl1 and Lhx3, suggesting the specificity of E1 (S2B Fig). GFP expression of mutE1-3 was completely lost even in the ventral spinal cord, indicating that E1-3 site activity relies on the endogenous Isl1 and Lhx3 present in motor neurons. MutE1-3 reporter activity was present in bm/vm neurons in the hindbrain when the reporter was introduced by electroporation, indicating that the E1-3 site is sm neuron-specific and its alteration by mutation does not affect basal transactivating ability (S6I and S6J Fig). To test whether the E1-3 site was sufficient to drive sm-specific gene expression, we generated a GFP reporter with six tandem repeats of the E1-3 site (6xE1-3). Expression of the 6xE1-3 reporter was selective in sm neurons by itself and expanded dorsally in the presence of Isl1 and Lhx3, indicating that Isl1-Lhx3 binding to the E1-3 site is sufficient to drive motor neuron-specific gene expression (Fig 6I, 6L and S4V Fig). Together our results suggest that Isl1-Lhx3 complex activates E1 in motor neurons.

### OC-1 binds to E2 enhancers to drive LMCm-specific expression of Isl1 in tetrapods

**Fig 7:**
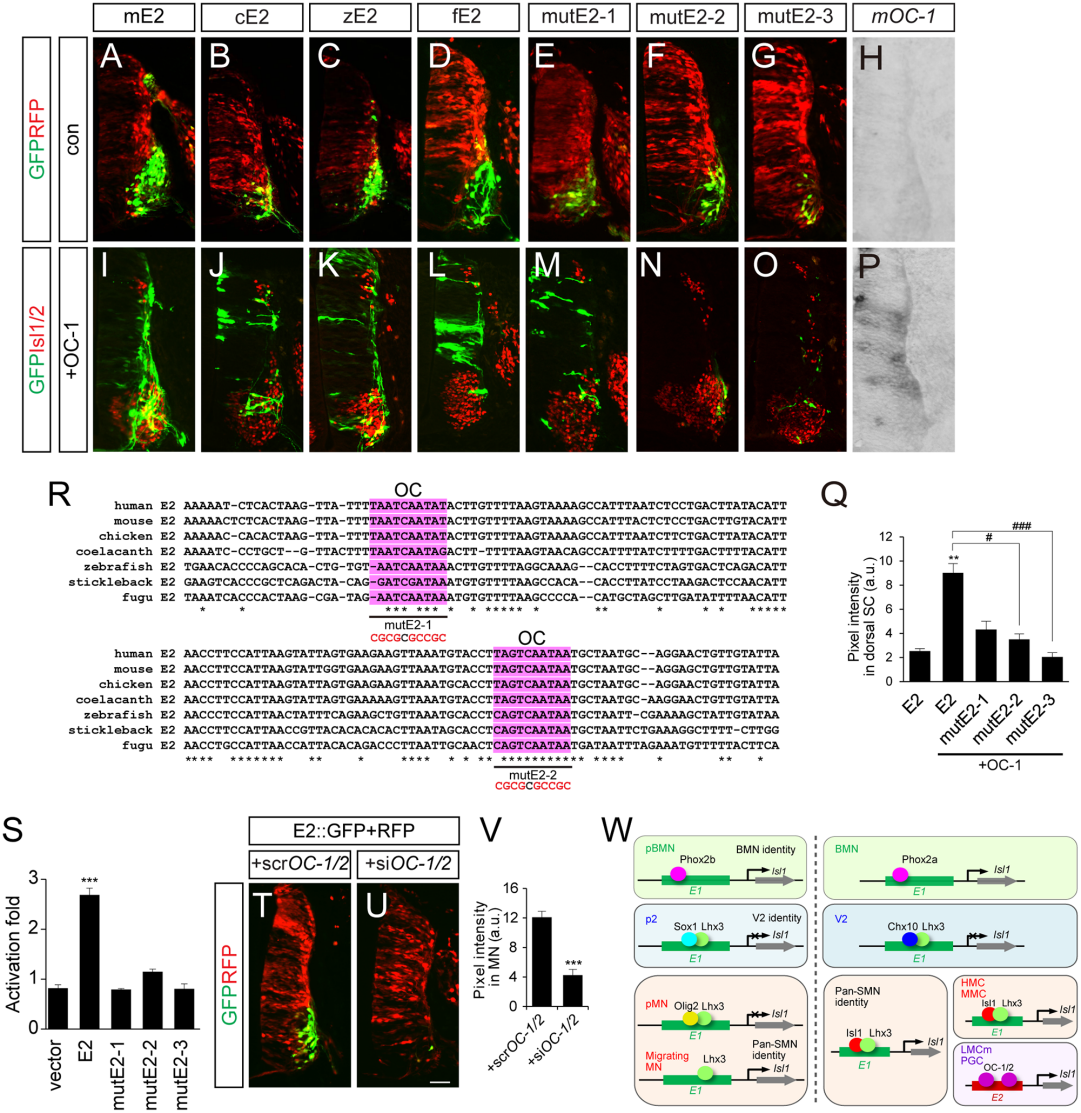
OC-1 activates the E2 enhancer in LMC neurons. (A-D, H, I-L, P) E2 GFP reporters from the mouse (mE2), chick (cE2), zebrafish (zE2) and fugu (fE2) were active in the chick spinal cord motor neurons and expanded their expression to the dorsal spinal cord in the presence of OC-1. Overexpression of mouse OC-1 was confirmed by in situ hybridization (P). (E-G, M-O) mutE2-3 carrying mutations in two OC-binding sites abolish responsiveness to OC-1, unlike mutE2-1 and mutE2-2, which have one site mutated. (Q) GFP intensity in dorsal spinal cord. Error bars represent SEM. **p < 0.01 vs. E1; #*p* < 0.05, ###*p* < 0.001 vs. E1 with OC; Kruskal-Wallis test (> 13 sections in 5 embryos in each group). (R) Multiple sequence alignment of potential OC-binding sites (shaded in pink) in the E2 enhancer in human, mouse, chicken, coelacanth, zebrafish, stickleback and fugu. Asterisks indicate conserved nucleotides conserved in all 7 species. Sequences for OC-1 motifs mutation assays were underlined and mutated sequences are shown in red. (S) Luciferase activity of E2 derivatives in the presence of OC-1. Error bars represent SEM. ***p < 0.001; unpaired Student’s t-test (n = 3). (T, U) Co-electroporation of si*OC-1* and si*OC-2* reduces E2::GFP reporter expression in the chick spinal cord. (V) Measurements of GFP intensity in each group. Error bar represents SEM. ***p < 0.001; unpaired Student’s t-test (n = 22 sections; *siOC-1* + si*OC-2*, n = 20 sections). (W) A model of arrangements of transcription factors and enhancers during motor neuron development. The diagram of Isl1-Lhx3 hexamer complex is simplified. Scale bar: 50 μm.

E2 sequences are conserved in mouse, chicken, zebrafish and fugu, but no E2 sequences were identifiable in the non-tetrapod chordate, lamprey and the cephalochordate amphioxus, at least in any currently available database [42–44] (S9 Fig). This indicates that E2 might have appeared in genomes when limb structures arise in the early vertebrates. All the E2 sequences derived from mouse, chicken and zebrafish were active in motor columns of the ventral spinal cord (Fig 7A–7C). We also generated putative E2 reporter from fugu and found that it was specific for motor neurons (Fig 7D). To search for E2-binding transcription factors, we first examined ChIP-Seq data for Phox2, Isl1 and Lhx3 binding in embryonic stem cell lines but found no significant peaks in the E2 region (S9 Fig) [31]. In a candidate approach, we decided to test the activity of OC factors, previously known to promote LMC identity and bind to E2 in ChIP assays [45]. When the OC-1 factor was co-electroporated, E2::GFP reporters became active in the entire spinal cord (Fig 7H–7L and 7P). OC-1 did not induce E1::luciferase reporter as previously reported (S2B Fig) [45]. Using the UCSC genome browser, we searched for two putative OC factor-binding sites in the E2 enhancer that were highly conserved in fugu, stickleback, zebrafish, coelacanth, chicken, mouse and human (Fig 7R). MutE2-1 and mutE2-2 retained their activity in motor neurons but only mutE2-1 responded to OC-1 (Fig 7E, 7F, 7H, 7M, 7N and 7P). When both sites were mutated in mutE2-3, the reporter was not induced by OC-1 (Fig 7G, 7O and 7Q). Similar responses were observed in luciferase assays in that the induction of E2 activity by OC-1 was significantly downregulated in mutE2-3 (Fig 7S). We also tested whether downregulation of OC factors affect E2 activity or not. When we knocked down OC-1 and OC-2 by siRNA, E2::GFP activity was significantly reduced (Fig 7T–7V and S7B–S7D Fig). Similarly, when we misexpressed Hoxc9 that switchs the LMC identity to thoracic motor neurons as determined by reduced expression of Foxp1 and *Raldh2*, E2 activity was also downregulated (S7E–S7L Fig) [46]. Together, our results suggest that OC factors are necessary and sufficient to induce the LMC-specific activity of the E2 enhancer.

### Evolutionary diversification of motor neuron-specific enhancers

**Fig 8:**
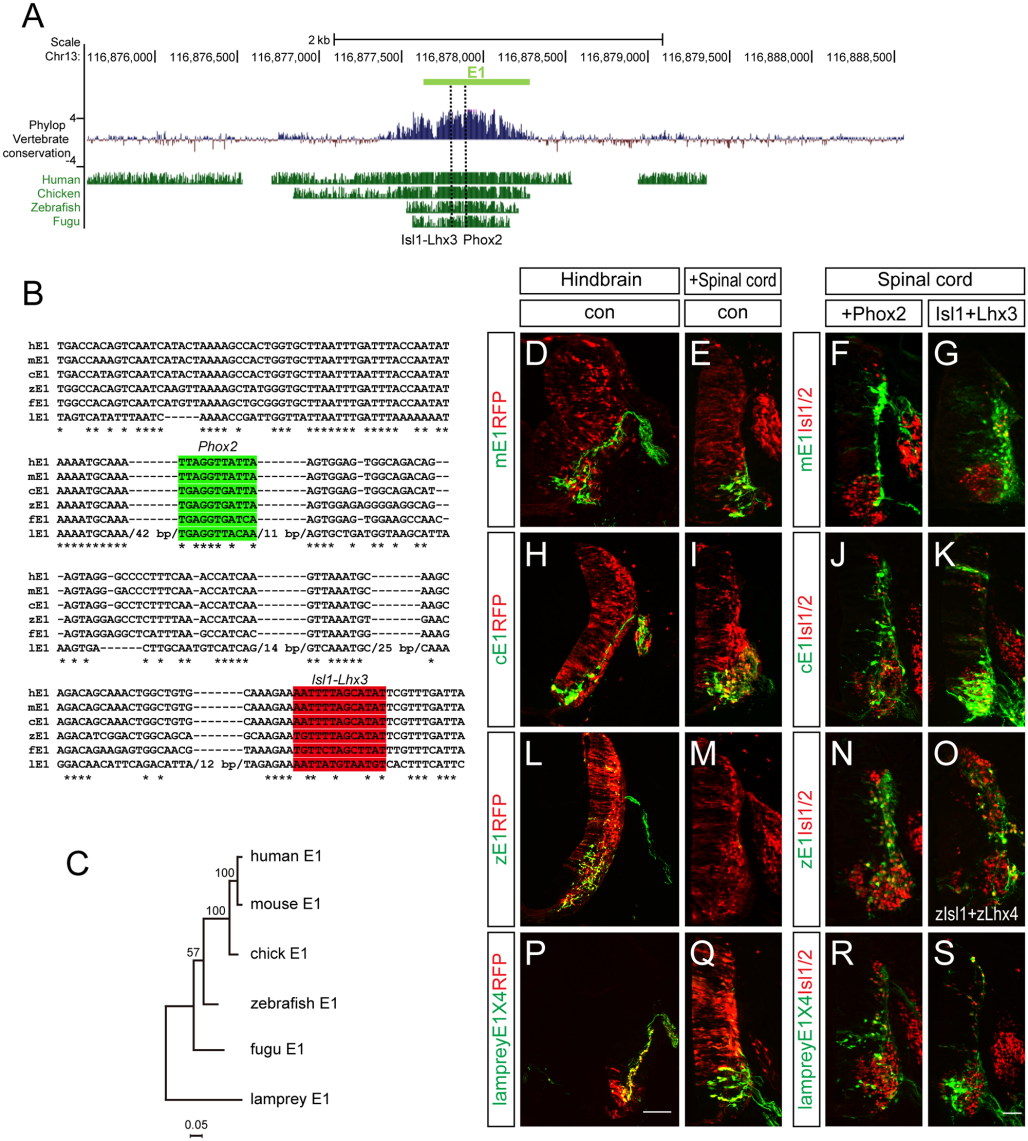
Conservation of putative regulatory elements of the E1 enhancer in vertebrates. (A) Alignment of the E1 enhancers in several organisms using the mouse E1 sequences as base line. Basewise conservation scores (phyloP) of vertebrate genomes (Human, Chicken, Zebrafish, Fugu) with mouse was shown. Dashed lines indicate Phox2 and Isl1-Lhx3 binding sites within E1. (B) Aligned Phox2 (shaded in green) and Isl1-Lhx3 (shaded in red) motif sequences in E1 enhancer among multiple species. Asterisks indicate conserved nucleotides conserved in all 7 species. Lamprey-specific insertion sites with > 10 bp are hidden with their full length base pair number. (C) A phylogenetic tree of E1. (D-S) The E1::GFP reporters from mouse, chick and zebrafish had different activities when electroporated into the chick neural tube. Scale bars: in P, 100 μm for D, H, L, P; in S, 50 μm for E-G, I-K, M-O, Q-S.

It has been shown that the E1 enhancer sequence is strongly conserved from fugu to human. We confirmed this, and found that the Phox2-binding and Isl1-Lhx3-binding motifs we identified were also highly conserved (Fig 8A). To examine the vertebrate origin of motor neurons, we tested for the presence of the E1 enhancer in the lamprey, a living representative of the most ancient vertebrates [42,43]. Although there was generally little conservation of the entire E1 sequences, we found that the core region of E1 containing the Phox2 and Isl1-Lhx3 binding sites were relatively well conserved (Fig 8B and 8C). To test whether enhancer activity was conserved, we generated GFP enhancers from the E1 sequences of mouse, chick, zebrafish and lamprey and electroporated them into the chick spinal cord. All the reporters showed bm neuron-specific GFP expression in the chick hindbrain (Fig 8D, 8H, 8L and 8P). The mouse, chick and lamprey E1 enhancers were active in the motor neurons of the spinal cord, and expanded in the presence of Phox2 or Isl1-Lhx3 (Fig 8E–8G, 8I–8K and 8Q–8S). However, zebrafish E1 was not active in the spinal cord motor neurons but nevertheless was induced by mPhox2 and zIsl1-zLhx4 in the dorsal spinal cord (Fig 8L–8O). The same analysis was applied to the E2 enhancer sequence. In contrast to Uemura et al, we detected the E2 enhancer sequence with conserved OC binding motifs in fugu [12]. We were unable to retrieve any putative E2 sequence from the lamprey genome when we conducted a BLAST search using E2 sequences from human, mouse, chicken, coelacanth, zebrafish, stickleback, and fugu [42]. We also found no match in amphioxus genomic data [44]. To be sure of the absence of E2 in the lamprey genome, we also analyzed vertebrate basewise conservation scores (phyloP) using the newest version, mmc10, which includes the lamprey genome (S9 Fig). Together, we conclude that motor neuron-specific E1 activity in the CNS is well-conserved from lamprey to man, whereas E2 appeared first in fugu along with the origin of limb/fin structures.

## Discussion

### The interplay between the E1 enhancer and transcription factors occurs in parallel with the diversification of hindbrain and spinal cord motor neurons

Motor neurons consist of multiple motor columns that innervate distinct muscle targets. Each motor column expresses some combination of transcription factors that form the basis for diversification of motor neurons [1,33]. Naturally the interplay between cis-regulatory elements (i.e., promoters and enhancers) and trans-regulatory elements (i.e., transcription factors) is critical for selective gene expression in individual motor neuronal subsets. In the present study, we focused on transcriptional regulation of *Islet1*, one of the representative markers of motor neurons that define motor neuron identity. Using stable and destabilized GFP reporters introduced into chick embryos, we demonstrated that E1 and E2 are differentially expressed in motor neuron subsets: E1 is active in all motor neurons and later becomes restricted to cranial motor neurons and MMC and PGC neurons in the spinal cord; E2 is more selective, being active in limb-innervating LMCm neurons that express Isl1, in line with previous reports in the zebrafish [12] (Fig 7W).

In the present work we have further characterized the transcription factors that interact with enhancers and found that Phox2 binds to E1 and drives *Isl1* expression in the hindbrain. Phox2a is necessary and sufficient to drive E1 activity since exogenous Phox2a induced E1 activity in embryos and cell lines, and knockdown of *Phox2b* by siRNA abrogated E1 activity. We designated as E1-4 the Phox2a binding site in E1 whose mutation abolished its activity in bm neurons but not in sm neurons. The mutE1-4 enhancer was not responsive to exogenous Phox2a but responded well to exogenous Isl1 and Lhx3, all of which supports the existence of bm neuron-specific gene transcription mechanisms. Similarly, we have demonstrated that the Isl1-Lhx3 complex induces E1 in sm neurons via the E1-3 site. Mutations at the E1-3 site abolished expression in sm neurons and prevented induction by Isl1-Lhx3. It is noteworthy that the mutE1-3 construct did not respond to exogenous Phox2a but was active in native bm neurons. Perhaps the cellular environment of the ectopic bm neurons was not as favorable as the native ones, and additional factors present in bona fide bm neurons may bind to this site and facilitate the action of Phox2a on E1. A likely candidate for such a factor is Isl1; the idea that it synergises with Phox2a is suggested by the surprisingly extensive overlap between binding sites for Phox2a and for Isl1 in a genomewide ChIP-Seq analysis [31]. Alternatively, Phox2a may act together with bHLH factors, as has been suggested to occur in sympathetic neurons [47].

Previous studies by us and others have demonstrated that Isl1 switches the stoichiometry of the tetrameric Lhx3 complex to that of a hexameric Isl1-Lhx3 complex to induce motor neuron identity, and aberrant assembly of the Lhx3 complex in motor neurons is prevented by repressors such as LMO factors and Hb9 [5,32,35]. In this study, we unexpectedly found that the Lhx3 complex is capable of activating E1, which may contribute to the initiation of *Isl1* transcription when motor neurons are about to become postmitotic. Nevertheless, ectopic expression of Isl1 in Lhx3-expressing motor neuron progenitors and V2 interneurons was blocked by the repressors Olig2, Sox1 and Chx10. These repressors were thus effective in blocking the activation of E1 by Lhx3. Interestingly, the ectopic formation of motor neurons and the E1 activity induced by Isl1-Lhx3 were also blocked by exogenous Chx10 but not by Olig2 and Sox1, suggesting that the potencies of these repressors or their mechanisms of action were different; repression may require DNA binding or protein-protein interaction. For instance, Olig2 may act as a repressor by binding to the E-box element, or it may squelch other bHLH factors such as Ngn2 by protein-protein interaction; either way it suppresses motor neuron formation [48]. In our study, we mapped the E-box site in E1 and found that mutating it made E1 insensitive to Olig2. Thus, DNA binding of Olig2 is important for its repressive action on E1. In the case of Chx10, mutating two consensus Chx10 binding sites had no effect. Interestingly, it is reported that the binding sites for Chx10 and hexameric Isl1-Lhx3 are similar and therefore Chx10 may inhibit Isl1-Lhx3 binding to the *Hb9* promoter [32]. In agreement with this we showed that Chx10 inhibits the transcriptional activation of tandem repeats of the E1-3 site by Isl1 and Lhx3. The point mutant Chx10 N51A deficient in DNA binding failed to inhibit the activity of E1-3 site, implying that Chx10 may bind to this site. What then would be the explanation for selective inhibition of Lhx3 complex activity by repressors? Our results in chick embryos and cell lines showed that the Lhx3 complex is less potent than the Isl1-Lhx3 complex in activating E1. This could be due to the lower DNA binding affinity of the Lhx3 complex demonstrated previously [32]. In addition, the binding elements for the Lhx3 and Isl1-Lhx3 complexes appear to be qualitatively different, and the different arrangements of multiple repeats and spacing between them should make the difference even greater, and may render the Lhx3 complex more susceptible to repressors [32].

It is noteworthy that Isl1 alone is not effective in inducing motor neuron identity or activating the E1, unlike Lhx3 or Isl1-Lhx3. Structure-function analysis suggests that NLI favors its binding to LIM domains of Lhx3 over Isl1, indicating that Lhx3 could be more efficient in forming a tetrameric complex in vivo [49]. In addition, the Lhx3 binding domain (LBD) of Isl1 at the C-terminus binds to Lhx3 to form hexameric Isl1-Lhx3 complex, which makes tetrameric Lhx3 or Isl1 complexes less available [5]. Recently, it is reported that the LBD domain also interacts with the LIM domains of Isl1 [50,51]. The intramolecular interaction is weak but specific, preventing unnecessary DNA binding and facilitating cofactor exchange in the cell. Indeed, Isl1 is known to act in synergy with other nuclear factors in neurons and other tissues [52–54]. Together, more diverse choice of cofactors and its tendency to suppress its own binding to DNA may explain weak biological activity of Isl1 by itself in motor neurons.

Considering the evolutionary emergence of transcription factors, it is noteworthy that Phox2 and Islet are found even in *Ciona internalis* in which Mnx-expressing somatic and Phox2-expressing visceral neurons are distinguishable [14,55]. Phox2 and Islet factors are also found in the lamprey and amphioxus, whose motor neurons are primitive, lacking motor columns and motor pools [56–58]. Thus, Phox2 and Isl1-Lhx3 were used in the motor neurons of ancient aquatic animals even before they diversified. In line with this, we identified Phox2 and Isl1-Lhx3 binding motifs in E1 enhancers from lamprey to man. Anatomical and genetic comparisons between species have suggested that the boundary between the hindbrain and spinal cord motor neurons is defined even in amphioxus (cephalochordate) and lamprey (chordate) [59–61]. Hence it appears that the motor neuron-specific activation of Isl1 with the help of Phox2 and Isl1-Lhx3 is evolutionarily conserved.

In this study, zebrafish E1 was not active in chick spinal cord motor neurons nor was it activated by exogenous Isl1-Lhx3 factors from other species. Nevertheless, we found that it was induced by zebrafish Isl1-Lhx4. There was only a one base difference between the Isl1-Lhx3 binding site in zE1 and the sites in mouse and chick. Substituting the binding site in mE1 for zE1 did not make it responsive to mIsl1 and mLhx3. It is possible that species differences in LIM-HD factors account for the absence of zE1 activity in chick spinal cords. However, the LIM-HD factors of zebrafish and chick differ by only one or two amino acids. In fact substantial cross-species variation has been reported in protein-DNA interactions that cannot be simply explained by changes in protein or DNA sequences [62,63]. Since an additional whole genome duplication event occurred in ray-finned fish including zebrafish, it is possible that extensive rearrangements of multiple cis- and trans-elements may have contributed to the zebrafish-specific transcription program.

### The E2 enhancer and OC factor define limb-innervating motor neurons in tetrapod animals

We and others have shown that the E2 enhancer is selective for LMCm neurons in the limb-innervating motor columns that express Isl1 [12]. Our detailed fate mapping analysis showed that E2 enhancer activity is strong in LMCm neurons. We and others also showed that E2 responded to OC-1 factors, which are important for LMC-specific gene expression [45]. In mice deficient in OC factors there are fewer LMCm neurons and more LMCl and PGC neurons as a result of a reduced level of Isl1 protein [45]. In line with this, we demonstrated that knockdown of OC factors or disruption of LMC identity by manipulating the *Hox* code coincided with reduced E2 activity. Taken together, these findings suggest that OC factors and E2 activity are important for maintaining Isl1 expression in LMCm neurons.

LMC neurons were added to motor columns when limb/fin structures appeared during evolution, and this coincided with the appearance of the Hox co-factor FoxP1 and loss of Lhx3 [16,19]. FoxP1 interacts with Hox proteins, which were altered in parallel with the emergence of paired fins/limbs [11,64]. Foxp1 also suppresses Lhx3, and this suppression is a prerequisite for LMC identity [10,11]. Likewise, in the absence of Foxp1, LMC neurons display HMC-like properties, reflecting their evolutionary origin [65]. Nevertheless, LMCm neurons maintain Isl1 expression in the absence of Lhx3, and thus in the absence of the Isl1-Lhx3 complex. Hence it is likely that tetrapod animals adopted the alternative enhancer E2 together with OC factors to maintain Isl1 expression in LMCm neurons due to the absence of Lhx3.

### Gradual specification of motor column identity

We used two reporters, one expressing stable GFP and the other, transient destabilized GFP, to trace cells that had activated the reporter at least once during their development and cells that were currently activating the enhancer, respectively. In this way, we expected to see whether motor column identity was acquired progressively or not. We found that the stable E1::GFP reporter marked all motor neurons, starting from the initial E1 activity in newborn motor neurons that had just begun to express Isl1. Thus, the transient assembly of Isl1 and Lhx3 in order to initially acquire motor neuron identity occurs in all motor neurons subsets, as previously suggested [5]. On the other hand, the destablized E1 reporter representing current E1 activity only labeled MMC and PGC. This suggests that E1 continues to be active in MMC and PGC, but that the initial activity of E1 disappears in the other motor columns. LMC neurons appear to initially use the E1 enhancer to obtain pan-motor neuron identity but later shut off E1 activity when they lose Lhx3. In the case of E2 reporters, the stable and transient E2 GFP reporters were both expressed in motor neurons once they reached a lateral position, mostly in LMCm neurons. Thus, the latter may use E1 and E2 sequentially to drive Isl1 expression during their transition from pan-motor neurons to LMC neurons. LMCl neurons acquire their identity when they pass through the LMCm area, in which they are exposed to retinoic acid signaling [66,67]. This induces Lhx1, which suppresses expression of Isl1 and specifies the LMCl fate. Thus, E2 may be initially active in all LMCs but later cease to be active in LMCl neurons when they migrate more laterally, with the help of Lhx1 and retinoids. Thus, gradual changes in the interactions between transcription factors and enhancers lead motor neurons to acquire their columnar identity progressively.

We have seen that E1 has been evolutionarily conserved since the era of the lamprey, a jawless vertebrate which possesses primitive motor neuron [56]. Recent comparative genomic studies have demonstrated that the segmentation of hindbrain and spinal cord is conserved in the lamprey, and is under the control of patterning signals such as *Hox* clusters [60,64,68]. The hindbrain and spinal cord motor neuron-specific expression of Islet1 could be achieved by E1 in the lamprey as part of the patterning program. However, the E2 enhancer, which is restricted to limb-innervating motor neurons, appeared when animals such as fugu and tetrapods developed limbs. E2 evolved to cooperate with OC factors and drive Isl1 expression in LMC neurons. Thus, our observations demonstrate that evolutionary conserved enhancers drive motor column-specific gene expression during motor neuron development.

## Materials and Methods

### DNA constructs

*Isl1* enhancers were amplified by PCR with primers using genomic DNA from mouse, chick, zebrafish, fugu and lamprey (S1 Table). PCR fragments were subcloned into the following reporter vectors: pCS2 mini CMV-GFP, pCS2 mini CMV-luciferase and *tk*-luciferase reporter vector [69]. pCS2 mini CMV-GFP/luciferase contains a 60 bp TATA box and the transcription initiation site of the cytomegalovirus (CMV) promoter (GenBank accession no. X03922: nt 1090–1149). The mini CMV promoter and eGFP sequences were obtained by PCR amplification from pEGFP-N1 (Clontech). Mutations were introduced in E1 or Chx10 by PCR-based mutagenesis. Zebrafish and Fugu E2 DNAs were amplified from *Dario rerio* and *Fugu rubripes*, respectively.

### Chick electroporation

In ovo electroporation was performed as described previously [35]. In brief, about 1 μg/μl of DNA solution was electroporated into the chick spinal cord at Hamburger and Hamilton (HH) stages 12 and harvested at HH stages 23 to 29. In the case of OC-1, HH stage 14 embryos were used. For the hindbrain electroporation, DNA solution was injected into HH stage 8 to 10 embryonic neural tube anteriorward from the level of approximately the third somite as previously described [70]. Electroporation was performed with a condition of 5 times of pulses, 20–24 volts, 50 msec, 1 sec intervals.

### Immunohistochemistry and *in situ* hybridization

Immunohistochemistry was performed as described previously [35]. Following antibodies were used: mouse anti-Isl1 (DSHB), mouse anti-Isl2 (DSHB), rabbit anti-Isl1/2 [22], mouse anti-MNR2 (DSHB), rabbit anti-GFP (Invitrogen), rabbit anti-Foxp1 (Abcam), rabbit anti-Sox1 (Cell signaling), guinea pig anti-Lhx3 [71], guinea pig anti-Chx10 [71], guinea pig anti-Olig2 [36] and mouse anti-Neurofilament (DSHB). For wholemount immunostaining, Day 4.5 chick embryos electroporated at Day 2 were fixed and incubated with primary antibodies for 3 days and secondary antibodies for 1 day. For *in situ* hybridization, transverse sections were hybridized with digoxigenin-labeled probes specific for mouse *Phox2a* (full CDS), mouse *OC-1* (partial CDS, 469–1263 bp). All images were captured with epifluorescent microscope or confocal microscope (Zeiss).

### Cell quantification

Cells in each quadrant of the ventral horn were counted on z-series of slice images using a confocal microscope and ZEN2009 imaging software (Zeiss). At least 3 embryos were quantified from each group, and 3–4 images were collected from each spinal cords. %GFP/column was calculated as the percentage of the number of GFP-expressing cells among the motor neurons in each column. To quantify GFP intensity induced by exogenous transcription factors in the chick spinal cords after electroporation, 12 μm-thick transverse sections were immunolabeled with GFP. The background-subtracted pixel intensities of GFP in 120 x 240 μm^2^ areas in the dorsal spinal cord were measured using ImageJ. At least 10 sections from 4 embryos were analyzed for each group. Statistical significance was analyzed by unpaired Student’s t-test and the Kruskal-Wallis test for multiple comparisons.

### Transgenic analysis

E1::GFP transgenic mice were generated in CL56BL6 background as described previously [72]. MutE1-4::GFP transgenes were prepared and linearized with the SalI/EcoRI for microinjection as described [73]. Transient transgenic embryos were generated by pronuclear injection into fertilized eggs. Animal experiments were performed under the guidelines of the Korea Ministry of Food and Drug Safety (Act No. 9025) with approval of procedures by the Institutional Animal Care and Use Committee of Gwangju Institute of Science and Technology (GIST-2010-12).

### Luciferase assays

293T cells were transiently transfected with reporters and transcription factors using Lipofectamine 2000 reagent (Invitrogen). CMV-β-galactosidase plasmid was co-transfected to normalize transfection efficiency. After 2 days, cell extracts were assayed for luciferase assays and β-galactosidase assays. Data represent as means of triplicate values, repeated at least three times.

### Bioinformatic analysis

Enhancer sequences from human, mouse, chicken, zebrafish, and fugu were retrieved from UCSC genome browser. Comparative analyses of the E1 and E2 sequences were done with mVISTA (genome.lbl.gov/vista) using the LAGAN alignment tool [74,75]. All E1 and E2 sequences from five species were searched against the lamprey genome using BLAST with “somewhat similar sequences (blastn)” options and an E-value cut-off of 0.05. Potential transcription factor binding sites in these conserved sequences were predicted using rVISTA [76]. A phylogenetic tree was constructed with MEGA version 6 maximum likelihood method with 1,000 bootstrap replications [77].

### Electrophoretic mobility shift assays

The sequences of sense and antisense oligonucleotide used in our EMSA were as follows:

E1-S 5′- CCAATATAAAATGCAAATTAGGTTATTAAGTGGAGTGGCAGAC-3′

E1-AS 5′-GTCTGCCACTCCACTTAATAACCTAATTTGCATTTTATATTGG-3′

mutE1-4-S 5′-CCAATATAAAATGCAACGCGGGTTCGCGAGTGGAGTGGCAGAC-3′

mutE1-4- AS ′-GTCTGCCACTCCACTCGCGAACCCGCGTTGCATTTTATATTGG-3′

Biotin-labeled probe was incubated with nuclear extract of HEK 293T cells transfected with mouse Phox2a in binding buffer (10 mM Tris, pH 7.5, 50 mM KCl, 5 mM MgCl_2_, 1 mM dithiothreitol, 0.05% Nonidet P-40, and 2.5% glycerol) with poly(dI-dC) at RT. Competition reactions were performed by adding a 200-fold excess of unlabeled double-stranded probe. The reactions were resolved on non-denaturing 6% polyacrylamide gels and visualized by chemiluminescence (Thermo Scientific).

### Chromatin immunoprecipitation

Embryonic mouse hindbrains were micro-dissected at E13.5. Cells were lysed after cross-linking with 1% formaldehyde. Chromatin with a DNA fragment length of less than 500 bp was obtained by sonication and immunoprecipitated with rabbit anti-Phox2b [78], rabbit anti-Histone H3 (Millipore) and rabbit IgG (Vector Labs). The Phox2 binding motif in E1 was amplified by PCR in each sample. For ChIP-Seq analysis, previous results were retrieved and examined around the *Isl1* locus [31].

## Supporting Information

S1 Fig Activity of the E1 enhancer fused with miniCMV or the minimal promoter of *Isl1* (*Isl1* mini P). (A-E) The miniCMV or *Isl1* mini P reporter alone does not promote GFP expression. Motor neuron-specific GFP expression occurs only when it is fused with the E1 enhancer. GFP activity of reporter with inverted E1 sequence is also motor neuron-specific (C). Scale bar: 50 μm
(TIF)

S2 Fig A search for transcription factors that activate the E1 enhancer. (A) Various candidate factors were transfected with the E1 luciferase reporter in 293T cells and luciferase activity was measured. Only Phox2a and Lhx3 induced reporter activity. (B) Among E1 and E2, Phox2a and Isl1-Lhx3 activate E1, and OC-1 activates E2. Error bar represents SEM using three replicates. *p < 0.05, **p < 0.01, ***p < 0.001; unpaired Student’s t-test (n = 3).
(TIF)

S3 Fig ChIP-Seq peaks around *Isl1* gene locus in NIP cells and NIL cells. Phox2a ChIP-Seq peaks (in NIP cells) and Isl1 and Lhx3 ChIP-Seq peaks (in NIL cells) around *Isl1* locus. Basewise conservation scores (phyloP) of vertebrate genomes (Human, Chicken, Zebrafish, Fugu) with mouse was shown.
(TIF)

S4 Fig Phox2 factors induce E1::GFP expression in the dorsal neural tube. (A-O) Phox2a induces the E1::GFP reporter in the dorsal spinal cord of HH24 chick embryos when introduced by electroporation, in which ectopic Isl1-expressing cells appeared (brackets, F, G, K, L). The adjacent sections show that the bm/vm neuron marker Tbx20 (bracket, H, M) was induced in the ectopic Isl1^+^ cells whereas the dI3 marker Brn3a and the sm neuron marker MNR2 (I, J, N, O), were not. E1mutE1-4 failed to induce GFP activity in the cells in which Isl1 was induced (bracket, K). ((P-X) Comparison of E1::GFP reporter derivatives using in ovo chick electroporation. CMV::RFP was co-electroporated as an internal control. The 320–479 E1 reporter was not active in the spinal cord (Q) but was active in the hindbrain (X). (Y) GFP pixel intensity in spinal cord motor neurons (SC MN). Error bar represents SEM using three replicates. ***p < 0.001; unpaired Student’s t-test (> 10 sections in 4 embryos in each group). Scale bars: in O, 50 μm for A-O; in X, 50 μm for P-X.
(TIF)

S5 Fig Summary of point mutations introduced in E1, and identification of Olig2 and Chx10 binding sites in E1. (A) Major binding motifs are highlighted and point mutations in the E1 sequence are underlined. The primer sequences used to generate the mutants are described in S1 Table. (B-F) Induction of E1 GFP reporter activity by Isl1 and Lhx3 is repressed in the presence of Chx10 but not by Olig2 and Sox1. (G-J) Lhx3-drived induction of E1 GFP is suppressed by Sox1 and ΔNSox1 but not by C-Sox1. (K-M) GFP expression from the mutE1-5 reporter is specific for motor neurons and is induced by Lhx3 or Isl1-Lhx3 in chick neural tubes when introduced by electroporation. This expression cannot be repressed by Olig2 when E1-5 is mutated. (N-S) Expression of GFP in mutE1-6 and mutE1-7 is induced by Lhx3 or Isl1-Lhx3, is inhibited by Chx10. (T-Y) Induction of 6xE1-3 by Lhx3 or Isl1-Lhx3 is blocked by Chx10 but not by the DNA-binding defective point mutant Chx10 N51A. Scale bar: 50 μm
(TIF)

S6 Fig The E1::GFP and mutE1-4::GFP activity in chicken embryos after electroporation. (A, B) Wholemount view of E4 chick embryos electroporated with the E1::GFP, mutE1-4::GFP and CMV::RFP at E2 as indicated. Both hindbrain and spinal cord were electroporated sequentially in the same embryo. Note that the mutE1-4::GFP lost its activity only in the hindbrain. (C-H’) Transverse sections of electroporated hindbrains. E1::GFP is found in both dorsally projecting-branchiomotor (filled arrowhead, C) and ventrally projecting-somatic motor axons (empty arrowhead, E). MutE1-4::GFP is only found in somatic motor axons (empty arrowhead, F). Both E1 and mutE1-4 reporters overlap with somatic motor neuronal marker MNR2 (empty arrowheads, G’, H’) (G-H’). (I, J) Transverse sections of chick hindbrains electroporated with E1::GFP and mutE1-3::GFP. MutE1-3 reporter maintained GFP expression in bm/vm neurons (filled arrowhead, J). Scale bars: in B, 1 mm for A, B; in D, 50 μm for C, D; in J, 100 μm for E-J
(TIF)

S7 Fig Knockdown of Phox2 and OC activity using siRNA in the chick neural tube. (A) In ovo RNAi using siRNA against chick *Phox2b* reduces the level of endogenous *Phox2b* transcripts at the site of electroporation (arrowhead). (B) HA-tagged chick *OC-1* and siRNA against scrambled (scr*OC-1*) or OC-1 (si*OC-1*) were transfected into 293T cells. Western blot analysis of cell lysates showed that HA expression was downregulated in the presence of si*OC-1*. (C, D) Knockdown efficiency of si*OC-1* and si*OC-2* was assessed by chick electroporation. si*OC-1* reduces the level of OC-1 protein (C) and si*OC-2* diminishes *OC-2* transcripts (D) in the electroporated side (right). (E-L) E2::GFP reporter activity was downregulated when the acquisition of LMC identity was inhibited by mHoxc9 (I), as shown by reduced expression of Foxp1 and *Raldh2* on the electroporated sides (J, L). Scale bars: in A, 100 μm; in D, 100 μm for C, D; in L, 100 μm for E-L.
(TIF)

S8 Fig Induction of the E1 reporter by Lhx3 is mediated by the LIM domain. (A-D) The E1::GFP reporter was not activated in chick embryos receiving ΔL-Lhx3, and Lhx3 with Lim-only protein 4 (LMO4) or the dimerization domain of NLI (DD) [1]. LMO4 and DD were expected to compete with Lhx3 in a LIM domain-specific manner. (E) GFP pixel intensity in dorsal spinal cord in each group. Error bar represents SEM using three replicates. **p < 0.01, ***p < 0.001; unpaired Student’s t-test (> 10 sections in 4 embryos in each group). (F) The E1 luciferase reporter is also induced by Lhx3 but not by ΔL-Lhx3 or Lhx3+DD. Error bar represents SEM using three replicates. **p < 0.01; unpaired Student’s t-test (n = 3). Scale bar: 100 μm.
(TIF)

S9 Fig ChIP-Seq peaks for Phox2, Isl1 and Lhx3 binding around E2. Phox2a ChIP-Seq peaks (in NIP cells) and Isl1 and Lhx3 ChIP-Seq peaks (in NIL cells) around *Crest2* (E2) locus. Basewise conservation scores (phyloP) of vertebrate genomes (Human, Chicken, Zebrafish, Fugu, Lamprey) with mouse was shown. Note that Lamprey genome does not have E2 (red shaded box).
(TIF)

S1 Table Primers used to amplify E1 and E2 derivatives. (XLSX)

S1 Movie 3D reconstruction of z-stack images of wholemount chick embryos electroporated with the E1::GFP reporter. Embryos were immunostained for GFP (green) and neurofilament (red). Images were collected in 12 z steps (approximately 15 μm interval) using a confocal microscope (Zeiss), and the 3D images were reconstructed by the Carl Zeiss Zen 2009 imaging software.
(MP4)

S2 Movie 3D reconstruction of z-stack images of wholemount chick embryos electroporated with the E2::GFP reporter. Embryos were immunostained for GFP (green) and neurofilament (red). Conditions for imaging and 3D reconstruction are described in S1 Movie.
(MP4)

S1 Text Supplemental Methods and Supplemental References. (DOCX)

## References

[1] Jessell TM (2000) Neuronal specification in the spinal cord: inductive signals and transcriptional codes. Nature Reviews Genetics 1: 20–29. 11262869 10.1038/35049541

[2] Dubreuil V, Hirsch M, Pattyn A, Brunet J, Goridis C (2000) The Phox2b transcription factor coordinately regulates neuronal cell cycle exit and identity. Development 127: 5191–5201. 11060244 10.1242/dev.127.23.5191

[3] Pattyn A, Hirsch M, Goridis C, Brunet J-F (2000) Control of hindbrain motor neuron differentiation by the homeobox gene Phox2b. Development 127: 1349–1358. 10704382 10.1242/dev.127.7.1349

[4] Brunet J-F, Pattyn A (2002) Phox2 genes-from patterning to connectivity. Current Opinion in Genetics & Development 12: 435–440. 12100889 10.1016/s0959-437x(02)00322-2

[5] Thaler JP, Lee S-K, Jurata LW, Gill GN, Pfaff SL (2002) LIM factor Lhx3 contributes to the specification of motor neuron and interneuron identity through cell-type-specific protein-protein interactions. Cell 110: 237–249. 12150931 10.1016/s0092-8674(02)00823-1

[6] Dasen J, Jessell T (2009) Hox networks and the origins of motor neuron diversity. Current topics in developmental biology 88: 169–200. 10.1016/S0070-2153(09)88006-X 19651305

[7] Alexander T, Nolte C, Krumlauf R (2009) Hox genes and segmentation of the hindbrain and axial skeleton. Annual Review of Cell and Developmental 25: 431–456. 10.1146/annurev.cellbio.042308.113423 19575673

[8] Philippidou P, Dasen J (2013) Hox Genes: Choreographers in Neural Development, Architects of Circuit Organization. Neuron 80: 12–34. 10.1016/j.neuron.2013.09.020 24094100 PMC3835187

[9] Pfaff SL, Mendelsohn M, Stewart CL, Edlund T, Jessell TM (1996) Requirement for LIM Homeobox Gene Isl1 in Motor Neuron Generation Reveals a Motor Neuron–Dependent Step in Interneuron Differentiation. Cell 84: 309–320. 8565076 10.1016/s0092-8674(00)80985-x

[10] Rousso D, Gaber Z, Wellik D, Morrisey E, Novitch B (2008) Coordinated actions of the forkhead protein Foxp1 and Hox proteins in the columnar organization of spinal motor neurons. Neuron 59: 226–240. 10.1016/j.neuron.2008.06.025 18667151 PMC2547125

[11] Dasen JS, De Camilli A, Wang B, Tucker PW, Jessell TM (2008) Hox repertoires for motor neuron diversity and connectivity gated by a single accessory factor, FoxP1. Cell 134: 304–316. 10.1016/j.cell.2008.06.019 18662545

[12] Uemura O, Okada Y, Ando H, Guedj M, Higashijima S, et al (2005) Comparative functional genomics revealed conservation and diversification of three enhancers of the isl1 gene for motor and sensory neuron-specific expression. Developmental biology 278: 587–606. 15680372 10.1016/j.ydbio.2004.11.031

[13] Kappen C, Salbaum JM (2009) Identification of regulatory elements in the Isl1 gene locus. The International journal of developmental biology 53: 935–946. 10.1387/ijdb.082819ck 19598113 PMC3482124

[14] Dufour HD, Chettouh Z, Deyts C, de Rosa R, Goridis C, et al (2006) Precraniate origin of cranial motoneurons. Proceedings of the National Academy of Sciences 103: 8727–8732. 10.1073/pnas.0600805103 PMC1482646 16735475

[15] Sugahara F, Aota S-i, Kuraku S, Murakami Y, Takio-Ogawa Y, et al (2011) Involvement of Hedgehog and FGF signalling in the lamprey telencephalon: evolution of regionalization and dorsoventral patterning of the vertebrate forebrain. Development 138: 1217–1226. 10.1242/dev.059360 21343370

[16] Fetcho JR (1992) The spinal motor system in early vertebrates and some of its evolutionary changes. Brain, behavior and evolution 40: 82–97. 1422809 10.1159/000113905

[17] Kusakabe R, Kuratani S (2005) Evolution and developmental patterning of the vertebrate skeletal muscles: perspectives from the lamprey. Developmental dynamics 234: 824–834. 16252276 10.1002/dvdy.20587

[18] Freitas R, Zhang G, Cohn MJ (2006) Evidence that mechanisms of fin development evolved in the midline of early vertebrates. Nature 442: 1033–1037. 16878142 10.1038/nature04984

[19] Funakoshi K, Nakano M (2007) The sympathetic nervous system of anamniotes. Brain, behavior and evolution 69: 105–113. 17230018 10.1159/000095199

[20] Osumi N, Hirota A, Ohuchi H, Nakafuku M, Iimura T, et al (1997) Pax-6 is involved in the specification of hindbrain motor neuron subtype. Development 124: 2961–2972. 9247338 10.1242/dev.124.15.2961

[21] Li X, Zhao X, Fang Y, Jiang X, Duong T, et al (1998) Generation of destabilized green fluorescent protein as a transcription reporter. Journal of Biological Chemistry 273: 34970–34975. 9857028 10.1074/jbc.273.52.34970

[22] Tsuchida T, Ensini M, Morton S, Baldassare M, Edlund T, et al (1994) Topographic organization of embryonic motor neurons defined by expression of LIM homeobox genes. Cell 79: 957–970. 7528105 10.1016/0092-8674(94)90027-2

[23] Stein S, Fritsch R, Lemaire L, Kessel M (1996) Checklist: vertebrate homeobox genes. Mechanisms of development 55: 91–108. 8734502 10.1016/0925-4773(95)00494-7

[24] Arber S, Han B, Mendelsohn M, Smith M, Jessell T, et al (1999) Requirement for the homeobox gene Hb9 in the consolidation of motor neuron identity. Neuron 23: 659–674. 10482234 10.1016/s0896-6273(01)80026-x

[25] Dasen J, Jessell T (2008) Hox networks and the origins of motor neuron diversity. Current topics in developmental biology 88: 169–200. 10.1016/S0070-2153(09)88006-X 19651305

[26] Hoffmann S, Berger IM, Glaser A, Bacon C, Li L, et al (2013) Islet1 is a direct transcriptional target of the homeodomain transcription factor Shox2 and rescues the Shox2-mediated bradycardia. Basic research in cardiology 108: 1–11. 10.1007/s00395-013-0339-z PMC3597335 23455426

[27] Francius C, Clotman F (2010) Dynamic expression of the Onecut transcription factors HNF-6, OC-2 and OC-3 during spinal motor neuron development. Neuroscience 165: 116–129. 10.1016/j.neuroscience.2009.09.076 19800948

[28] Sander M, Paydar S, Ericson J, Briscoe J, Berber E, et al (2000) Ventral neural patterning by Nkx homeobox genes: Nkx6. 1 controls somatic motor neuron and ventral interneuron fates. Genes & development 14: 2134–2139. 10970877 10.1101/gad.820400 PMC316892

[29] Ericson J, Rashbass P, Schedl A, Brenner-Morton S, Kawakami A, et al (1997) Pax6 controls progenitor cell identity and neuronal fate in response to graded Shh signaling. Cell 90: 169–180. 9230312 10.1016/s0092-8674(00)80323-2

[30] Song M-R, Shirasaki R, Cai C-L, Ruiz EC, Evans SM, et al (2006) T-Box transcription factor Tbx20 regulates a genetic program for cranial motor neuron cell body migration. Development 133: 4945–4955. 17119020 10.1242/dev.02694 PMC5851594

[31] Mazzoni EO, Mahony S, Closser M, Morrison CA, Nedelec S, et al (2013) Synergistic binding of transcription factors to cell-specific enhancers programs motor neuron identity. Nature neuroscience 16: 1219–1227. 10.1038/nn.3467 23872598 PMC3820498

[32] Lee S, Lee B, Joshi K, Pfaff SL, Lee JW, et al (2008) A regulatory network to segregate the identity of neuronal subtypes. Developmental cell 14: 877–889. 10.1016/j.devcel.2008.03.021 18539116 PMC3071743

[33] Shirasaki R, Pfaff SL (2002) Transcriptional codes and the control of neuronal identity. Annual review of neuroscience 25: 251–281. 12052910 10.1146/annurev.neuro.25.112701.142916

[34] Hutchinson SA, Eisen JS (2006) Islet1 and Islet2 have equivalent abilities to promote motoneuron formation and to specify motoneuron subtype identity. Development 133: 2137–2147. 16672347 10.1242/dev.02355

[35] Song M-R, Sun Y, Bryson A, Gill GN, Evans SM, et al (2009) Islet-to-LMO stoichiometries control the function of transcription complexes that specify motor neuron and V2a interneuron identity. Development 136: 2923–2932. 10.1242/dev.037986 19666821 PMC2723064

[36] Novitch BG, Chen AI, Jessell TM (2001) Coordinate regulation of motor neuron subtype identity and pan-neuronal properties by the bHLH repressor Olig2. Neuron 31: 773–789. 11567616 10.1016/s0896-6273(01)00407-x

[37] Zhou Q, Anderson DJ (2002) The bHLH transcription factors OLIG2 and OLIG1 couple neuronal and glial subtype specification. Cell 109: 61–73. 11955447 10.1016/s0092-8674(02)00677-3

[38] Genethliou N, Panayiotou E, Panayi H, Orford M, Mean R, et al (2009) SOX1 links the function of neural patterning and Notch signalling in the ventral spinal cord during the neuron-glial fate switch. Biochemical and biophysical research communications 390: 1114–1120. 10.1016/j.bbrc.2009.08.154 19723505

[39] Karunaratne A, Hargrave M, Poh A, Yamada T (2002) GATA proteins identify a novel ventral interneuron subclass in the developing chick spinal cord. Developmental biology 249: 30–43. 12217316 10.1006/dbio.2002.0754

[40] Kan L, Israsena N, Zhang Z, Hu M, Zhao L, et al (2004) Sox1 acts through multiple independent pathways to promote neurogenesis. Developmental biology 269: 580–594. 15110721 10.1016/j.ydbio.2004.02.005

[41] Dorval KM, Bobechko BP, Fujieda H, Chen S, Zack DJ, et al (2006) CHX10 targets a subset of photoreceptor genes. Journal of Biological Chemistry 281: 744–751. 16236706 10.1074/jbc.M509470200

[42] Smith JJ, Kuraku S, Holt C, Sauka-Spengler T, Jiang N, et al (2013) Sequencing of the sea lamprey (Petromyzon marinus) genome provides insights into vertebrate evolution. Nature genetics 45: 415–421. 10.1038/ng.2568 23435085 PMC3709584

[43] McEwen GK, Goode DK, Parker HJ, Woolfe A, Callaway H, et al (2009) Early evolution of conserved regulatory sequences associated with development in vertebrates. PLoS genetics 5: e1000762 10.1371/journal.pgen.1000762 20011110 PMC2781166

[44] Putnam NH, Butts T, Ferrier DE, Furlong RF, Hellsten U, et al (2008) The amphioxus genome and the evolution of the chordate karyotype. Nature 453: 1064–1071. 10.1038/nature06967 18563158

[45] Roy A, Francius C, Rousso DL, Seuntjens E, Debruyn J, et al (2012) Onecut transcription factors act upstream of Isl1 to regulate spinal motoneuron diversification. Development 139: 3109–3119. 10.1242/dev.078501 22833130 PMC4074251

[46] Jung H, Lacombe J, Mazzoni EO, Liem KF Jr, Grinstein J, et al (2010) Global Control of Motor Neuron Topography Mediated by the Repressive Actions of a Single Hox Gene. Neuron 67: 781–796. 10.1016/j.neuron.2010.08.008 20826310 PMC2955411

[47] Vincentz JW, VanDusen NJ, Fleming AB, Rubart M, Firulli BA, et al (2012) A Phox2-and Hand2-dependent Hand1cis-regulatory element reveals a unique gene dosage requirement for Hand2 during sympathetic neurogenesis. The Journal of Neuroscience 32: 2110–2120. 10.1523/JNEUROSCI.3584-11.2012 22323723 PMC3324095

[48] Lee S-K, Lee B, Ruiz EC, Pfaff SL (2005) Olig2 and Ngn2 function in opposition to modulate gene expression in motor neuron progenitor cells. Genes & Development 19: 282–294. 15655114 10.1101/gad.1257105 PMC545894

[49] Gadd MS, Bhati M, Jeffries CM, Langley DB, Trewhella J, et al (2011) Structural basis for partial redundancy in a class of transcription factors, the LIM homeodomain proteins, in neural cell type specification. Journal of Biological Chemistry 286: 42971–42980. 10.1074/jbc.M111.248559 22025611 PMC3234805

[50] Gadd MS, Jacques DA, Nisevic I, Craig VJ, Kwan AH, et al (2013) A structural basis for the regulation of the LIM-homeodomain protein Islet 1 (Isl1) by intra-and intermolecular interactions. Journal of Biological Chemistry 288: 21924–21935. 10.1074/jbc.M113.478586 23750000 PMC3724647

[51] Sanchez-Garcia I, Osada H, Forster A, Rabbitts T (1993) The cysteine-rich LIM domains inhibit DNA binding by the associated homeodomain in Isl-1. The EMBO journal 12: 4243–4250. 7901000 10.1002/j.1460-2075.1993.tb06108.x PMC413719

[52] Liu J, Hunter CS, Du A, Ediger B, Walp E, et al (2011) Islet-1 Regulates Arx Transcription during Pancreatic Islet α-Cell Development. The Journal of Biological Chemistry 286: 15352–15360. 10.1074/jbc.M111.231670 21388963 PMC3083195

[53] Zhang H, Wang W, Guo T, Yang J, Chen P, et al (2009) The LIM-homeodomain protein ISL1 activates insulin gene promoter directly through synergy with BETA2. Journal of molecular biology 392: 566–577. 10.1016/j.jmb.2009.07.036 19619559

[54] Nasif S, de Souza F, González L, Yamashita M, Orquera D, et al (2015) Islet 1 specifies the identity of hypothalamic melanocortin neurons and is critical for normal food intake and adiposity in adulthood. Proceedings of the National Academy of Sciences of the United States of America 112: E1861–1870. 10.1073/pnas.1500672112 25825735 PMC4403183

[55] Giuliano P, Marino R, Pinto MR, De Santis R (1998) Identification and developmental expression of Ci-isl, a homologue of vertebrate islet genes, in the ascidian Ciona intestinalis. Mechanisms of development 78: 199–202. 9858732 10.1016/s0925-4773(98)00143-9

[56] Fetcho JR (1987) A review of the organization and evolution of motoneurons innervating the axial musculature of vertebrates. Brain Research Reviews 12: 243–280. 10.1016/0165-0173(87)90001-4 3300861

[57] Fetcho J, Reich N (1992) Axial motor organization in postmetamorphic tiger salamanders (Ambystoma tigrinum): a segregation of epaxial and hypaxial motor pools is not necessarily associated with terrestrial locomotion. Brain, behavior and evolution 39: 219–228. 1633553 10.1159/000114119

[58] Jackman W, Langeland J, Kimmel C (2000) islet Reveals Segmentation in the Amphioxus Hindbrain Homolog. Developmental Biology 220: 16–26. 10720427 10.1006/dbio.2000.9630

[59] Murakami Y, Pasqualetti M, Takio Y, Hirano S, Rijli FM, et al (2004) Segmental development of reticulospinal and branchiomotor neurons in lamprey: insights into the evolution of the vertebrate hindbrain. Development 131: 983–995. 14973269 10.1242/dev.00986

[60] Parker HJ, Bronner ME, Krumlauf R (2014) A Hox regulatory network of hindbrain segmentation is conserved to the base of vertebrates. Nature 514: 490–493. 10.1038/nature13723 25219855 PMC4209185

[61] Holland LZ, Holland ND (1999) Chordate origins of the vertebrate central nervous system. Current opinion in neurobiology 9: 596–602. 10508734 10.1016/S0959-4388(99)00003-3

[62] Odom DT, Dowell RD, Jacobsen ES, Gordon W, Danford TW, et al (2007) Tissue-specific transcriptional regulation has diverged significantly between human and mouse. Nature genetics 39: 730–732. 17529977 10.1038/ng2047 PMC3797512

[63] Ritter DI, Li Q, Kostka D, Pollard KS, Guo S, et al (2010) The importance of being cis: evolution of orthologous fish and mammalian enhancer activity. Molecular biology and evolution 27: 2322–2332. 10.1093/molbev/msq128 20494938 PMC3107594

[64] Jung H, Mazzoni EO, Soshnikova N, Hanley O, Venkatesh B, et al (2014) Evolving Hox Activity Profiles Govern Diversity in Locomotor Systems. Developmental Cell 29: 171–187. 10.1016/j.devcel.2014.03.008 24746670 PMC4024207

[65] Murakami Y, Tanaka M (2011) Evolution of motor innervation to vertebrate fins and limbs. Developmental biology 355: 164–172. 10.1016/j.ydbio.2011.04.009 21540022

[66] Eberhart J, Swartz M, Koblar SA, Pasquale E, Krull CE (2002) EphA4 constitutes a population-specific guidance cue for motor neurons. Developmental biology 247: 89–101. 12074554 10.1006/dbio.2002.0695

[67] Kania A, Jessell TM (2003) Topographic motor projections in the limb imposed by LIM homeodomain protein regulation of ephrin-A: EphA interactions. Neuron 38: 581–596. 12765610 10.1016/s0896-6273(03)00292-7

[68] Mehta TK, Ravi V, Yamasaki S, Lee AP, Lian MM, et al (2013) Evidence for at least six Hox clusters in the Japanese lamprey (Lethenteron japonicum). Proceedings of the National Academy of Sciences 110: 16044–16049. 10.1073/pnas.1315760110 PMC3791769 24043829

[69] Lee S-K, Jurata LW, Funahashi J, Ruiz EC, Pfaff SL (2004) Analysis of embryonic motoneuron gene regulation: derepression of general activators function in concert with enhancer factors. Development 131: 3295–3306. 15201216 10.1242/dev.01179

[70] Yasugi S, Nakamura H (2000) Gene transfer into chicken embryos as an effective system of analysis in developmental biology. Development, growth & differentiation 42: 195–197. 10.1046/j.1440-169x.2000.00500.x 10910123

[71] Thaler JP, Koo SJ, Kania A, Lettieri K, Andrews S, et al (2004) A postmitotic role for Isl-class LIM homeodomain proteins in the assignment of visceral spinal motor neuron identity. Neuron 41: 337–350. 14766174 10.1016/s0896-6273(04)00011-x

[72] Shirasaki R, Lewcock JW, Lettieri K, Pfaff SL (2006) FGF as a target-derived chemoattractant for developing motor axons genetically programmed by the LIM code. Neuron 50: 841–853. 16772167 10.1016/j.neuron.2006.04.030

[73] Lee B, Rizzoti K, Kwon DS, Kim S-Y, Oh S, et al (2012) Direct transcriptional regulation of Six6 is controlled by SoxB1 binding to a remote forebrain enhancer. Developmental biology 366: 393–403. 10.1016/j.ydbio.2012.04.023 22561201 PMC4009495

[74] Brudno M, Do CB, Cooper GM, Kim MF, Davydov E, et al (2003) LAGAN and Multi-LAGAN: efficient tools for large-scale multiple alignment of genomic DNA. Genome research 13: 721–731. 12654723 10.1101/gr.926603 PMC430158

[75] Frazer KA, Pachter L, Poliakov A, Rubin EM, Dubchak I (2004) VISTA: computational tools for comparative genomics. Nucleic acids research 32: W273–W279. 15215394 10.1093/nar/gkh458 PMC441596

[76] Loots GG, Ovcharenko I (2004) rVISTA 2.0: evolutionary analysis of transcription factor binding sites. Nucleic acids research 32: W217–W221. 15215384 10.1093/nar/gkh383 PMC441521

[77] Tamura K, Stecher G, Peterson D, Filipski A, Kumar S (2013) MEGA6: molecular evolutionary genetics analysis version 6.0. Molecular biology and evolution 30: 2725–2729. 10.1093/molbev/mst197 24132122 PMC3840312

[78] Pattyn A, Morin X, Cremer H, Goridis C, Brunet J-F (1997) Expression and interactions of the two closely related homeobox genes Phox2a and Phox2b during neurogenesis. Development 124: 4065–4075. 9374403 10.1242/dev.124.20.4065

