# Supplementary figures and images for: Functional Diversification of Motor Neuron-specific Isl1 Enhancers during Evolution
Source: PLoS Genet. 2015 Oct 8;11(10):e1005560. doi: 10.1371/journal.pgen.1005560 (PMC4598079; doi:10.1371/journal.pgen.1005560)

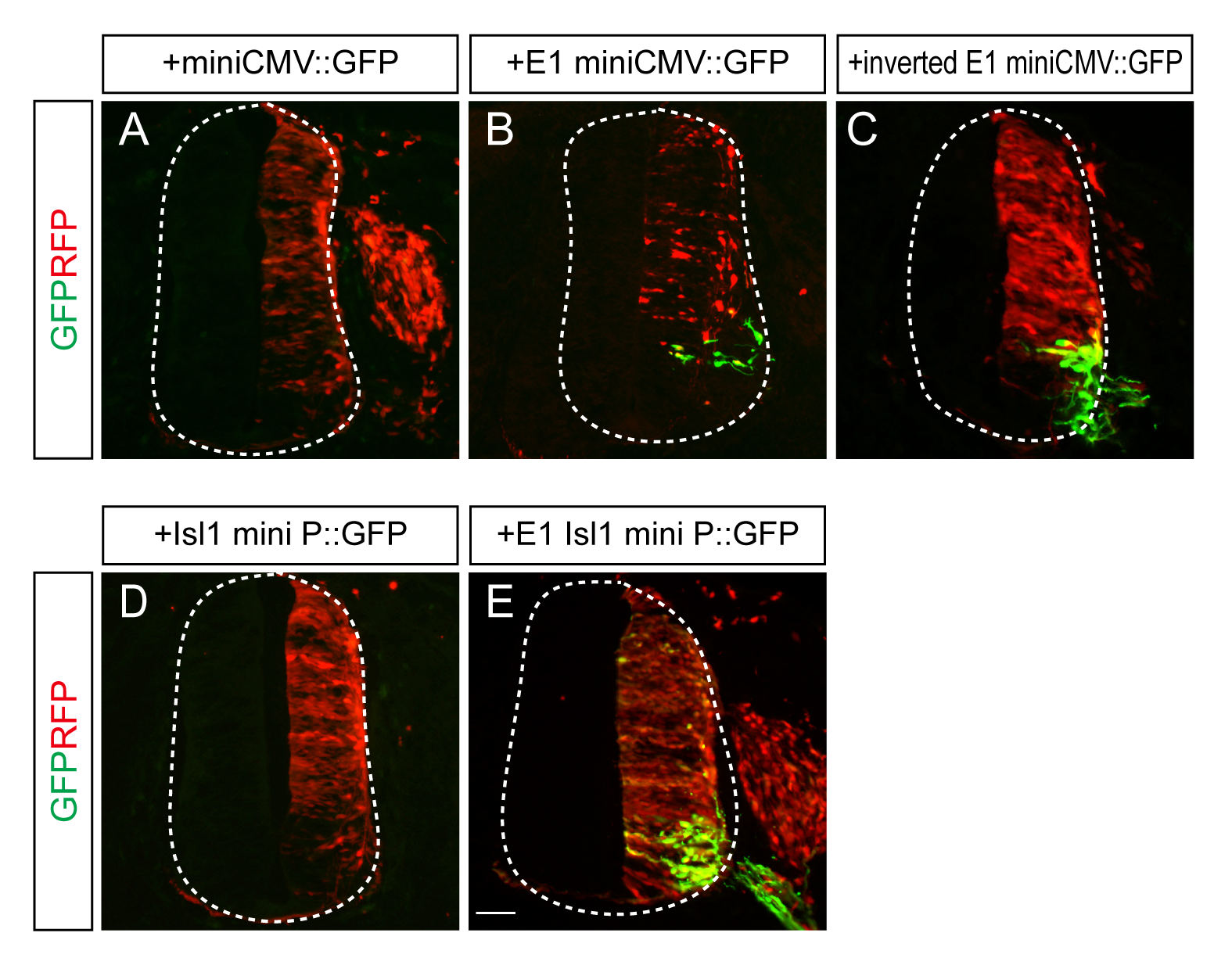

Supplement: S1 Fig — (A-E) The miniCMV or Isl1 mini P reporter alone does not promote GFP expression. Motor neuron-specific GFP expression occurs only when it is fused with the E1 enhancer. GFP activity of reporter with inverted E1 sequence is also motor neuron-specific (C). Scale bar: 50 μm (TIF) [file pgen.1005560.s001.tif]

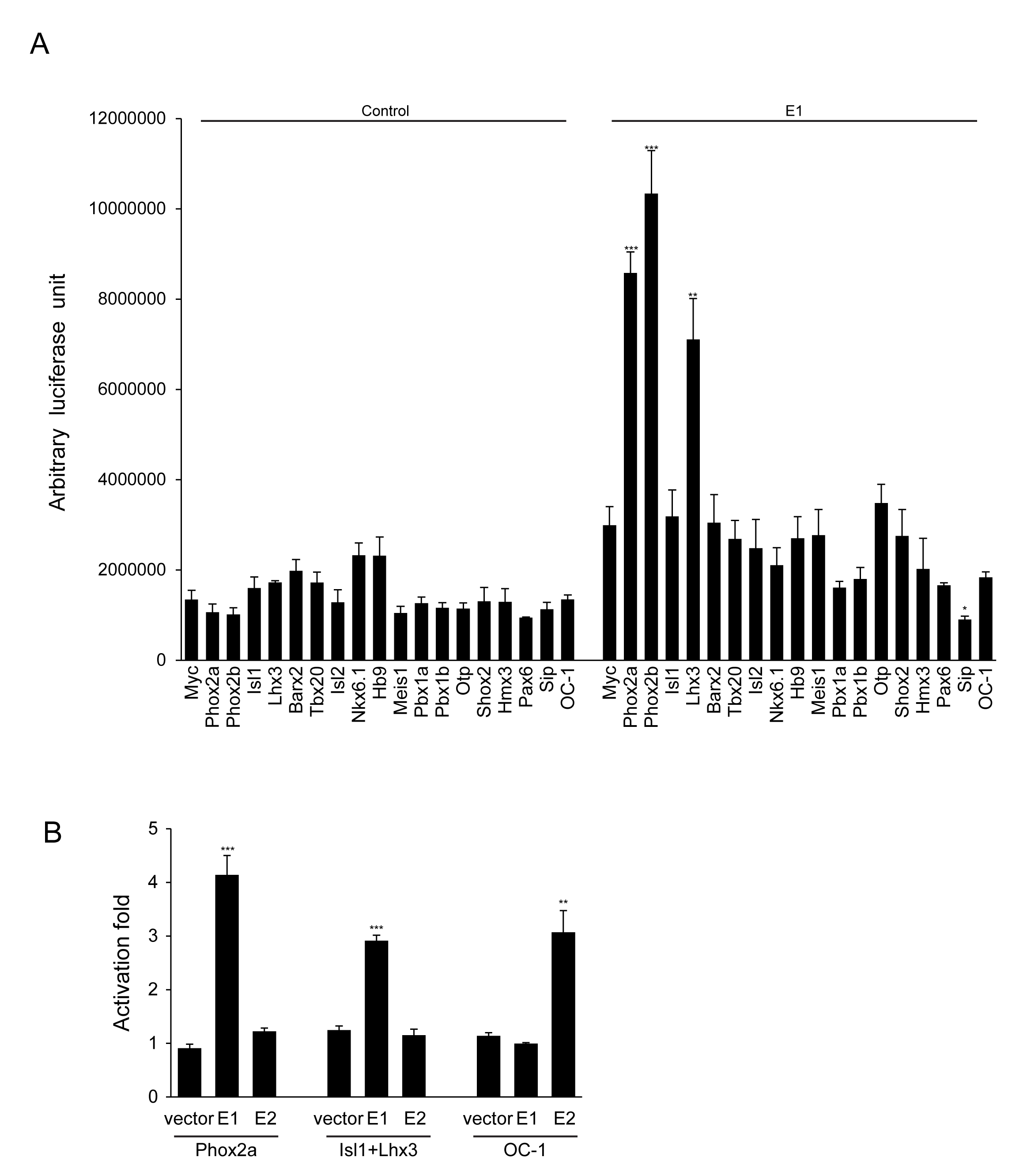

Supplement: S2 Fig — (A) Various candidate factors were transfected with the E1 luciferase reporter in 293T cells and luciferase activity was measured. Only Phox2a and Lhx3 induced reporter activity. (B) Among E1 and E2, Phox2a and Isl1-Lhx3 activate E1, and OC-1 activates E2. Error bar represents SEM using three replicates. *p < 0.05, **p < 0.01, ***p < 0.001; unpaired Student’s t-test (n = 3). (TIF) [file pgen.1005560.s002.tif]

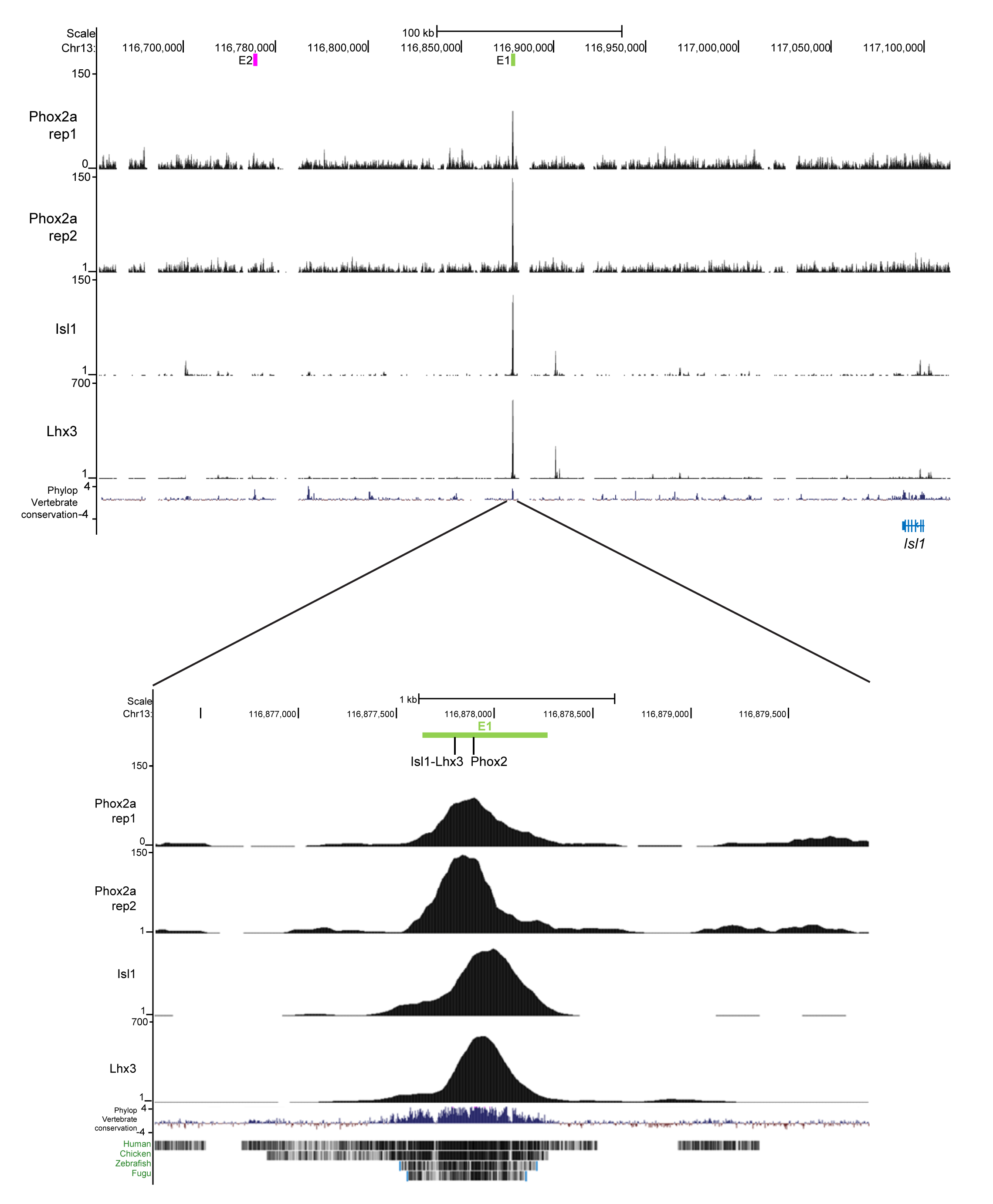

Supplement: S3 Fig — Phox2a ChIP-Seq peaks (in NIP cells) and Isl1 and Lhx3 ChIP-Seq peaks (in NIL cells) around Isl1 locus. Basewise conservation scores (phyloP) of vertebrate genomes (Human, Chicken, Zebrafish, Fugu) with mouse was shown. (TIF) [file pgen.1005560.s003.tif]

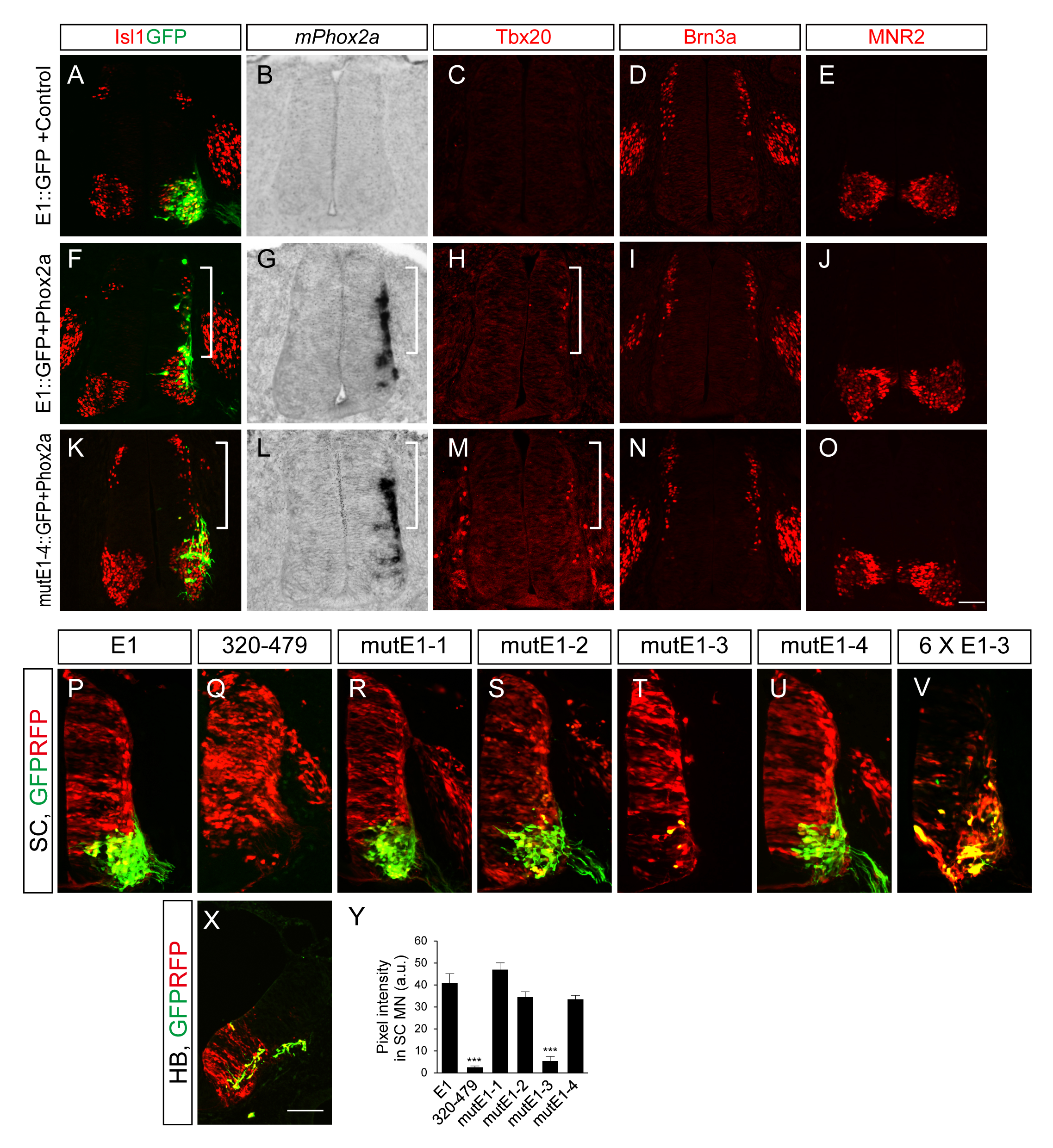

Supplement: S4 Fig — (A-O) Phox2a induces the E1::GFP reporter in the dorsal spinal cord of HH24 chick embryos when introduced by electroporation, in which ectopic Isl1-expressing cells appeared (brackets, F, G, K, L). The adjacent sections show that the bm/vm neuron marker Tbx20 (bracket, H, M) was induced in the ectopic Isl1+ cells whereas the dI3 marker Brn3a and the sm neuron marker MNR2 (I, J, N, O), were not. E1mutE1-4 failed to induce GFP activity in the cells in which Isl1 was induced (bracket, K). ((P-X) Comparison of E1::GFP reporter derivatives using in ovo chick electroporation. CMV::RFP was co-electroporated as an internal control. The 320–479 E1 reporter was not active in the spinal cord (Q) but was active in the hindbrain (X). (Y) GFP pixel intensity in spinal cord motor neurons (SC MN). Error bar represents SEM using three replicates. ***p < 0.001; unpaired Student’s t-test (> 10 sections in 4 embryos in each group). Scale bars: in O, 50 μm for A-O; in X, 50 μm for P-X. (TIF) [file pgen.1005560.s004.tif]

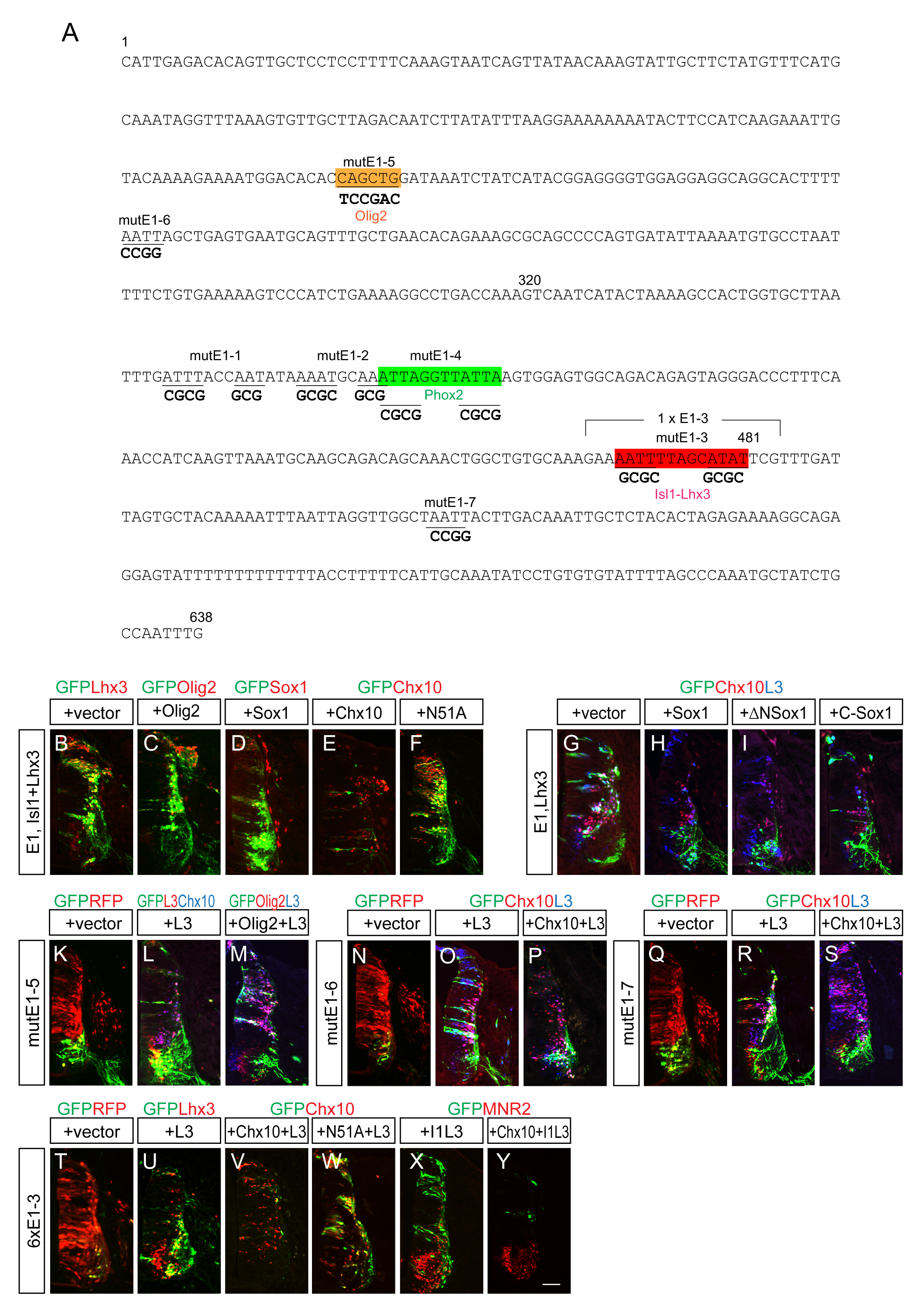

Supplement: S5 Fig — (A) Major binding motifs are highlighted and point mutations in the E1 sequence are underlined. The primer sequences used to generate the mutants are described in S1 Table. (B-F) Induction of E1 GFP reporter activity by Isl1 and Lhx3 is repressed in the presence of Chx10 but not by Olig2 and Sox1. (G-J) Lhx3-drived induction of E1 GFP is suppressed by Sox1 and ΔNSox1 but not by C-Sox1. (K-M) GFP expression from the mutE1-5 reporter is specific for motor neurons and is induced by Lhx3 or Isl1-Lhx3 in chick neural tubes when introduced by electroporation. This expression cannot be repressed by Olig2 when E1-5 is mutated. (N-S) Expression of GFP in mutE1-6 and mutE1-7 is induced by Lhx3 or Isl1-Lhx3, is inhibited by Chx10. (T-Y) Induction of 6xE1-3 by Lhx3 or Isl1-Lhx3 is blocked by Chx10 but not by the DNA-binding defective point mutant Chx10 N51A. Scale bar: 50 μm (TIF) [file pgen.1005560.s005.tif]

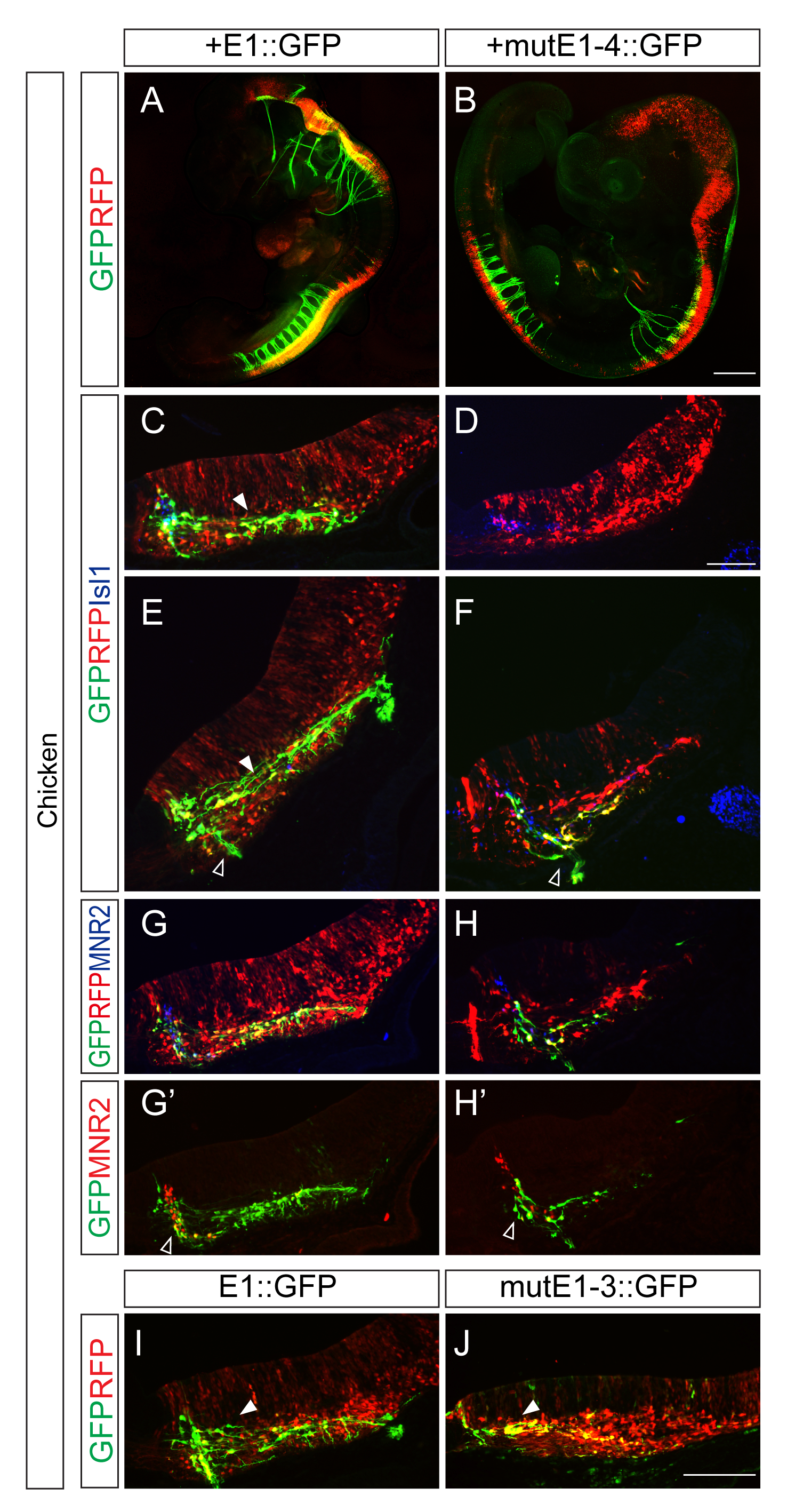

Supplement: S6 Fig — (A, B) Wholemount view of E4 chick embryos electroporated with the E1::GFP, mutE1-4::GFP and CMV::RFP at E2 as indicated. Both hindbrain and spinal cord were electroporated sequentially in the same embryo. Note that the mutE1-4::GFP lost its activity only in the hindbrain. (C-H’) Transverse sections of electroporated hindbrains. E1::GFP is found in both dorsally projecting-branchiomotor (filled arrowhead, C) and ventrally projecting-somatic motor axons (empty arrowhead, E). MutE1-4::GFP is only found in somatic motor axons (empty arrowhead, F). Both E1 and mutE1-4 reporters overlap with somatic motor neuronal marker MNR2 (empty arrowheads, G’, H’) (G-H’). (I, J) Transverse sections of chick hindbrains electroporated with E1::GFP and mutE1-3::GFP. MutE1-3 reporter maintained GFP expression in bm/vm neurons (filled arrowhead, J). Scale bars: in B, 1 mm for A, B; in D, 50 μm for C, D; in J, 100 μm for E-J (TIF) [file pgen.1005560.s006.tif]

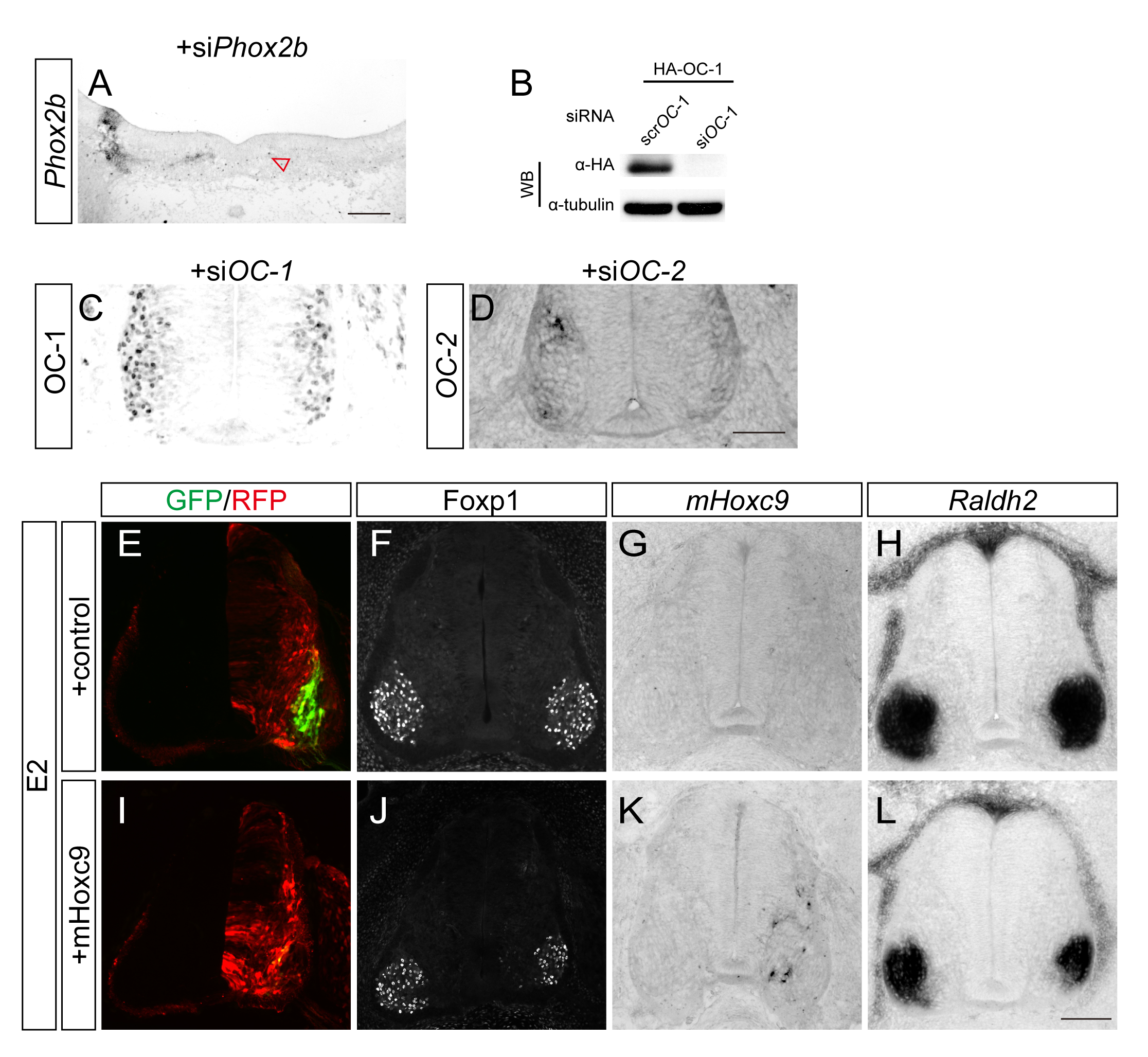

Supplement: S7 Fig — (A) In ovo RNAi using siRNA against chick Phox2b reduces the level of endogenous Phox2b transcripts at the site of electroporation (arrowhead). (B) HA-tagged chick OC-1 and siRNA against scrambled (scrOC-1) or OC-1 (siOC-1) were transfected into 293T cells. Western blot analysis of cell lysates showed that HA expression was downregulated in the presence of siOC-1. (C, D) Knockdown efficiency of siOC-1 and siOC-2 was assessed by chick electroporation. siOC-1 reduces the level of OC-1 protein (C) and siOC-2 diminishes OC-2 transcripts (D) in the electroporated side (right). (E-L) E2::GFP reporter activity was downregulated when the acquisition of LMC identity was inhibited by mHoxc9 (I), as shown by reduced expression of Foxp1 and Raldh2 on the electroporated sides (J, L). Scale bars: in A, 100 μm; in D, 100 μm for C, D; in L, 100 μm for E-L. (TIF) [file pgen.1005560.s007.tif]

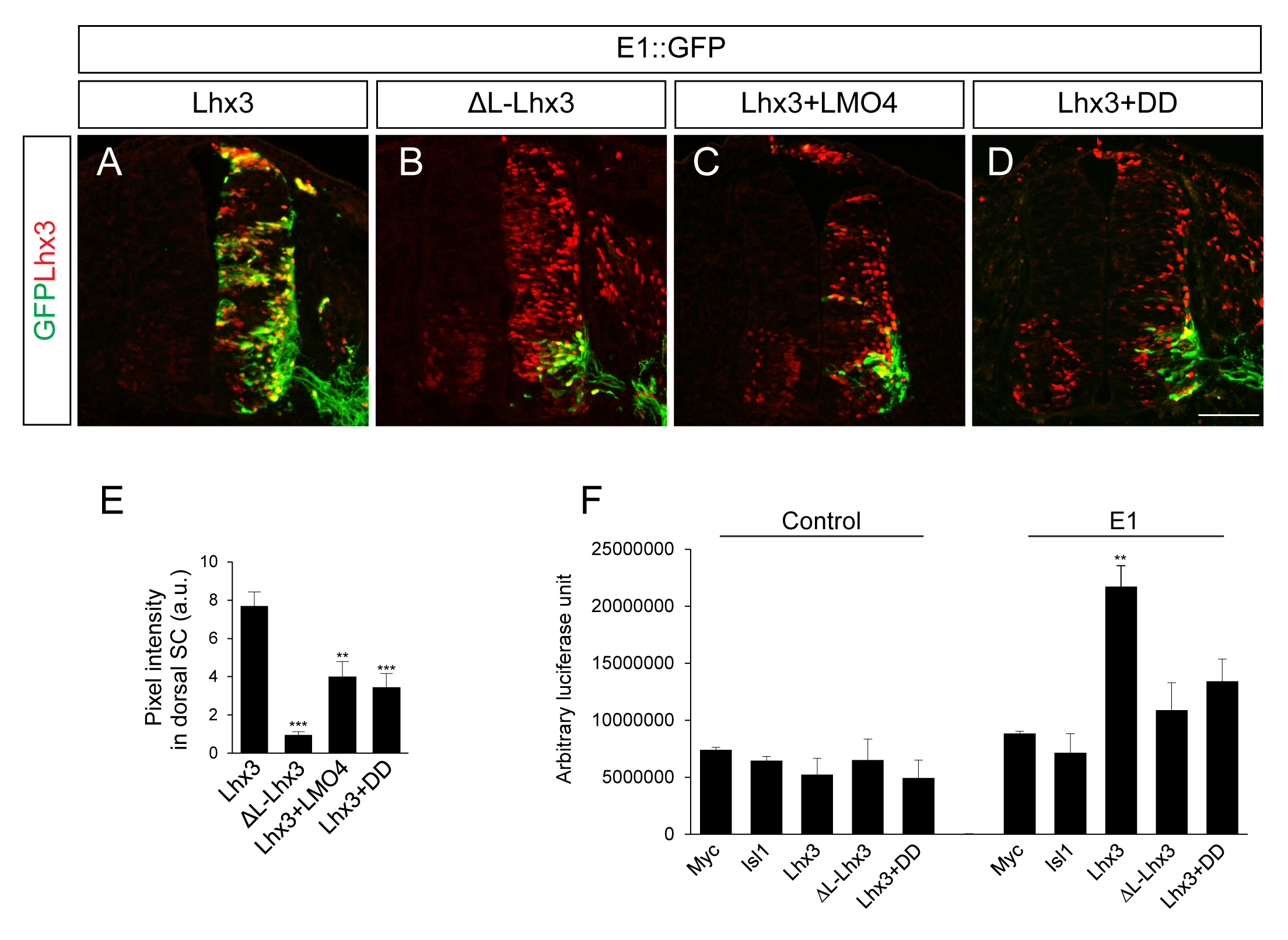

Supplement: S8 Fig — (A-D) The E1::GFP reporter was not activated in chick embryos receiving ΔL-Lhx3, and Lhx3 with Lim-only protein 4 (LMO4) or the dimerization domain of NLI (DD) [1]. LMO4 and DD were expected to compete with Lhx3 in a LIM domain-specific manner. (E) GFP pixel intensity in dorsal spinal cord in each group. Error bar represents SEM using three replicates. **p < 0.01, ***p < 0.001; unpaired Student’s t-test (> 10 sections in 4 embryos in each group). (F) The E1 luciferase reporter is also induced by Lhx3 but not by ΔL-Lhx3 or Lhx3+DD. Error bar represents SEM using three replicates. **p < 0.01; unpaired Student’s t-test (n = 3). Scale bar: 100 μm. (TIF) [file pgen.1005560.s008.tif]

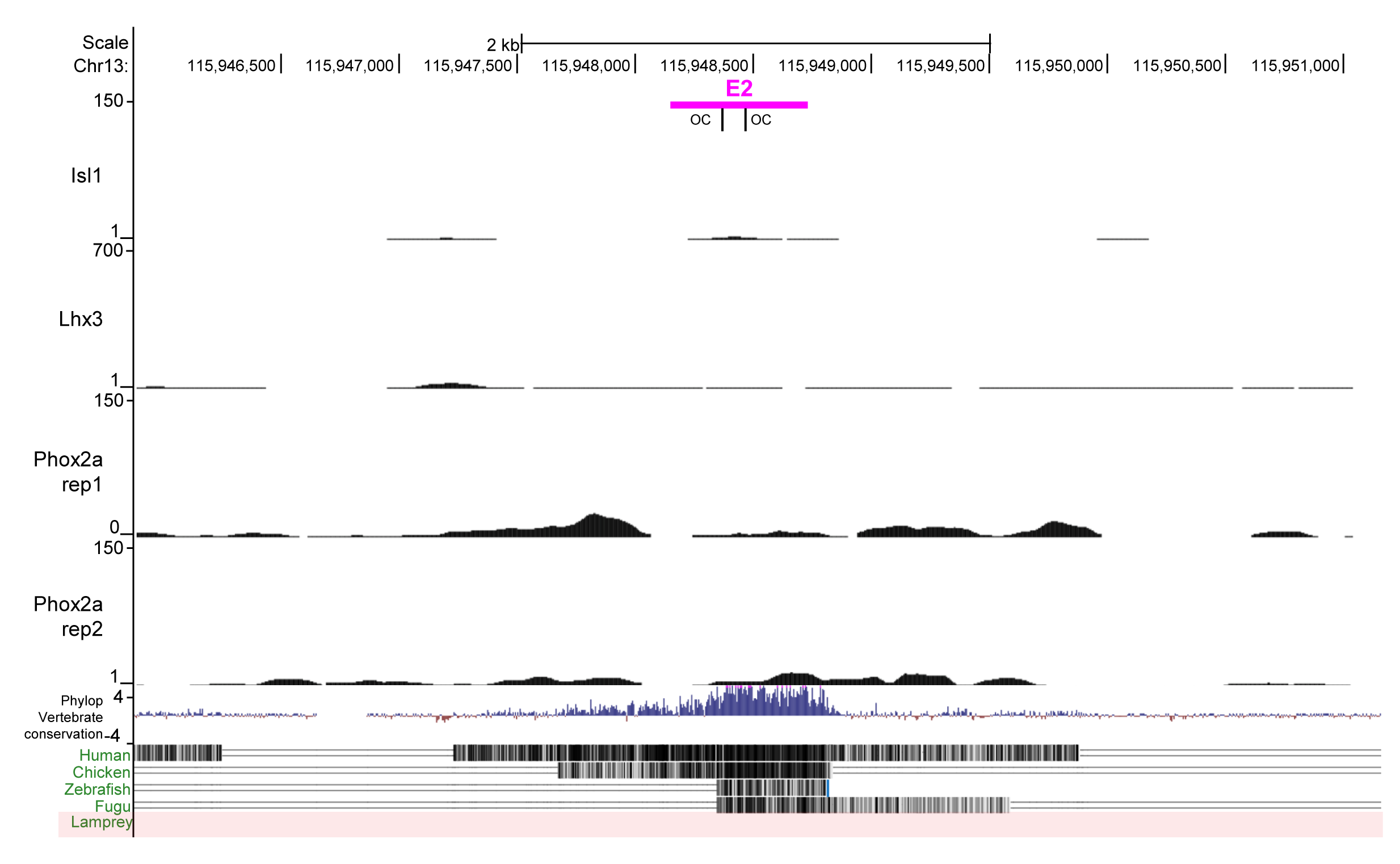

Supplement: S9 Fig — Phox2a ChIP-Seq peaks (in NIP cells) and Isl1 and Lhx3 ChIP-Seq peaks (in NIL cells) around Crest2 (E2) locus. Basewise conservation scores (phyloP) of vertebrate genomes (Human, Chicken, Zebrafish, Fugu, Lamprey) with mouse was shown. Note that Lamprey genome does not have E2 (red shaded box). (TIF) [file pgen.1005560.s009.tif]
