# Supplementary material for: Functional Diversification of Motor Neuron-specific Isl1 Enhancers during Evolution
Source: PLoS Genet. 2015 Oct 8;11(10):e1005560. doi: 10.1371/journal.pgen.1005560 (PMC4598079; doi:10.1371/journal.pgen.1005560)
Supplement: S1 Text — (DOCX) [file pgen.1005560.s013.docx]

**Supplemental Methods**

**DNA construct**

The sequence of miniCMV was described in Material and Methods. *Isl1* mini P was obtained by PCR amplification from mouse genomic DNA with primers: F-5′-TGATAATCAGAACAGCTGCG-3′, R- 5′-CCTTATCTCTTACTCAAACTT-3′. cDNAs used to test the activity of E1::luciferase assays were amplified from E10.5 mouse cDNAs. m*Hoxc9* and ck*Raldh2* were amplified from mouse and chick embryonic cDNA using Diastar OneStep RT-PCR kit (Solgent). Mutation in DNA binding domain of Chx10 (Chx10 N51A) was introduced by PCR based mutagenesis using Pfu DNA polymerase (Solgent). Follwing primers were used for point mutation:

F-5′-GGATACAGGTGTGGTTCCAGGCCCGCAGAGCCAAGTGGAGGA-3′,

R- 5′-TCCTCCACTTGGCTCTGCGGGCCTGGAACCACACCTGTATCC

ΔNSox1 (Sox1 without the N-terminus) and C-Sox1 (C-terminal portion of Sox1, lacking HMG box) were constructed by replacing the full-length Sox1 as previously reported [2].

The primers were as follows:

ΔNSox1: F-EcoR1-ΔNSox1: 5’- ataGAATTCCCCATGAACGCCTTCATGG - 3’, R-Xho1-ΔNSox1: 5’-ataCTCGAGCTAGATGTGCGTCAGGGGCA- 3’

C-Sox1: F-EcoR1- C-Sox1: 5’- ataGAATTCATGGACAAGTACTCGCTGGC- 3’, R-Xho1- C-Sox1: 5’- ataCTCGAGCTAGATGTGCGTCAGGGGCA - 3’

**RNA interference**

siRNA oligonucleotide duplexes targeting chick *Phox2b* [3], chick *OC-1* and *OC-2* were synthesized by Bioneer. 0.5 μg/μl of siRNAs and *Isl1* enhancer::GFP reporter were electroporated into chick spinal cords or hindbrains. The targeting sequences were as follows: si*Phox2b*: 5’-CAAGAACGGAGCGGCCGGCAA- 3’; scrambled *Phox2b*: 5’-GCG AGA CAC GAG ACG GCA ACG- 3’; si*OC-1*: 5’-CGGCCGAGCTGAAGCGTTA - 3’; scrambled *OC-1*:5’-ATCGGCGTGGCTAAGACCG- 3’; ck*OC-2*: 5’-GGAACAACTCCCAGAAGAA - 3’; scrambled *OC-2* :5’-ATGAACGACAGACAAGACC- 3’. Knockdown efficiency of si*Phox2b,* si*OC-1* and si*OC-2* were assessed by immunostaining or *in situ* hybridization after chick electroporation. Rabbit anti-OC-1 antibody (Santa Cruz) and digoxigenin-labeled RNA probes for chick *Phox2b* (partial CDS, 270-834 bp) and chick *OC-2* (3’UTR, 1748-2645 bp) were used. Knockdown efficiency of si*OC-1* was also tested by western blot analysis.

**Western blot analysis**

293T cells were transfected with HA-tagged chick OC-1 with siRNAs. Cell lysates were harvested and processed for Western blot analysis. Anti-HA (Covance) and anti-α-tubulin (AbD Serotec) antibodies were used.

**Supplemental References**

1. Thaler JP, Lee S-K, Jurata LW, Gill GN, Pfaff SL (2002) LIM factor Lhx3 contributes to the specification of motor neuron and interneuron identity through cell-type-specific protein-protein interactions. Cell 110: 237-249.
2. Kan L, Israsena N, Zhang Z, Hu M, Zhao L, et al. (2004) Sox1 acts through multiple independent pathways to promote neurogenesis. Developmental Biology 269: 580-594.
3. Reiff T, Tsarovina K, Majdazari A, Schmidt M, del Pino I, et al. (2010) Neuroblastoma phox2b variants stimulate proliferation and dedifferentiation of immature sympathetic neurons. Journal of Neuroscience 30: 905-915.
